# Optimized methods for measuring competitive binding of chemical substances to thyroid hormone distributor proteins transthyretin and thyroxine binding globulin

**DOI:** 10.1007/s00204-024-03842-y

**Published:** 2024-08-21

**Authors:** Yang Shen, Toine F. H. Bovee, Douwe Molenaar, Yoran Weide, Antsje Nolles, Carmen Braucic Mitrovic, Stefan P. J. van Leeuwen, Jochem Louisse, Timo Hamers

**Affiliations:** 1 Wageningen Food Safety Research (WFSR), Wageningen University and Research, Akkermaalsbos 2, 6708 WB Wageningen, The Netherlands; 2 Amsterdam Institute for Life and Environment (A-LIFE), Vrije Universiteit Amsterdam, De Boelelaan 1085, 1081 HV Amsterdam, The Netherlands; 3 Present Address: European Food Safety Authority (EFSA), Parma, Italy

**Keywords:** Thyroid hormone system disruptors, TTR binding, TBG binding, Fluorescence polarization, Per- and polyfluoroalkyl substances

## Abstract

Transthyretin (TTR) and thyroxine-binding globulin (TBG) are two major thyroid hormone (TH) distributor proteins in human plasma, playing important roles in stabilizing the TH levels in plasma, delivery of TH to target tissues, and trans-barrier transport. Binding of xenobiotics to these distributor proteins can potentially affect all these three important roles of distributor proteins. Therefore, fast and cost-effective experimental methods are required for both TTR and TBG to screen both existing and new chemicals for their potential binding. In the present study, the TTR-binding assay was therefore simplified, optimized and pre-validated, while a new TBG-binding assay was developed based on fluorescence polarization as a readout. Seven model compounds (including positive and negative controls) were tested in the pre-validation study of the optimized TTR-binding assay and in the newly developed TBG-binding assay. The dissociation constants of the natural ligand (thyroxine, T4) and potential competitors were determined and compared between two distributor proteins, showing striking differences for perfluorooctanesulfonic acid (PFOS) and perfluorooctanoic acid (PFOA).

**Supplementary Information:**

The online version contains supplementary material available at 10.1007/s00204-024-03842-y.

## Introduction

Thyroid hormone (TH) plays an important role in regulating energy metabolism, the development of the nervous system and brain, growth and differentiation, and renal function (Dellovade et al. 2000; Murk et al. 2013). Thyroxine (3,3′,5,5′-tetraiodothyronine or T4) is the main form of TH secreted by the thyroid gland and 3,3′,5-triiodothyronine (T3), which is generated by T4 deiodination, is the most biologically active form of TH. Some chemicals have been reported to affect TH homeostasis, and consequently may cause adverse effects. Thyroid hormone system dysfunction has been reported to adversely affect (fetal) neurodevelopment, cognitive function, and the cardiovascular system in adults (Kester et al. 1999). Therefore, thyroid hormone system disruptors (THSDs) are of increasing concern to the public and researchers, because such chemicals have been detected in environmental matrices and food samples, and may potentially lead to adverse effects to animals and humans (Boas et al. 2006; Coperchini et al. 2021; Freire et al. 2013; Zhao et al. 2021). THSDs may interfere with TH synthesis by disrupting its regulation through the hypothalamus–pituitary–thyroid (HPT) axis, by inhibiting iodide uptake into the thyroid gland, and/or by inhibiting the thyroperoxidase (TPO) enzyme responsible for oxidation of thyroglobulin. Alternatively, THSDs may interfere with TH distribution via competitive binding to TH distributor proteins TTR, TBG and albumin (ALB) in blood, with TH metabolism by induction and/or inhibition of deiodinase enzymes, dehalogenase enzymes, or phase-2 sulfotransferase and uridine 5'-diphospho-glucuronosyltransferase enzymes, with TH uptake in target cells by inhibiting TH transmembrane transport proteins, and with TH signalling in target cells by activating or inactivating TH receptors (Dong and Wade 2017; Köhrle and Frädrich 2021; Liu et al. 2015).

TTR, TBG and ALB are the three most important TH distributor proteins in blood (Alshehri et al. 2015). TTR is a 55 kD homotetramer and the molecule has two iodothyronine binding sites, which are deeply embedded in the protein (Hamilton and Benson 2001). Usually only one TTR binding site is occupied by a T4 molecule, as a negative cooperative effect greatly reduces the binding affinity of the second site (Refetoff 2023). TTR present in plasma is synthesized in the liver. In human blood, 15% of T4 is transported by TTR (Vieira and Saraiva 2014), while TTR is the main carrier of T4 in rodents (Palha et al. 1994). Previous in vivo studies linked competitive binding to TTR to decreased TH levels in rodents, due to increased hepatic clearance of unbound TH (Hallgren and Darnerud 2002; Liu et al. 2011). TTR is also produced by the choroid plexus and secreted to the cerebrospinal fluid (CSF) in both rodents and humans. TTR plays an important role in TH passage across the blood-CSF barrier (BCSFB) during the early stages of development in many species (Landers and Richard 2017). Moreover, TTR plays a vital role in transporting T4 across the placenta and T4 delivery to the fetus (Landers et al. 2009). In pregnant rats, decreased fetal plasma T4 levels were obtained after the exposure of the rats to hydroxylated polychlorinated biphenyls (OH-PCBs), which was expected to result from the competitive binding of OH-PCBs to TTR (Meerts et al. 2002). Currently, an adverse outcome pathway (AOP152) is under development (included in the OECD (Organisation for Economic Co-operation and Development) work plan) that includes binding of a chemical to TTR in the serum as the molecular initiating event (MIE), adversely impacting hippocampal anatomy, function, and ultimately, cognitive function (Janus et al. 2021). In addition, an AOP with competitive binding to TTR as MIE, ultimately leading to altered amphibian metamorphosis by affecting the levels of TH in serum, is included in the AOP-wiki (AOP366) (Haselma et al. 2021).

TBG is a 54 kD acidic glycoprotein and contains one T4 binding site in a surface pocket (Refetoff 2023). TBG is synthesized in the liver, and carries 75% of plasma T4 and T3 in humans (Pappa et al. 2015). In contrast to TTR, TBG is limitedly present in rats, and has been detected only postnatally (0–50 days) and in aged rats (7 months) (Savu et al. 1991). TBG was also reported to be synthesized in the choroid plexus and to be present in CSF in humans, though the level was two orders of magnitude lower than that of TTR (Hagen and Elliott 1973). Available information of TBG pathophysiology indicates that the deficiency or excess of TBG results in an abnormal serum T4 level in humans (Chakravarthy and Ejaz 2019). Furthermore, a cross-sectional study suggested that decreased total T4 levels associated with exposure to PCB105 in premenopausal females and PCB153 in males aged < 50, may be explained by a decrease in TBG (Kim et al. 2022).

Given the physiological functional importance of both TTR and TBG, it is necessary to develop test methods to screen chemicals for their potential to affect TH homeostasis by competing with T4 for binding to its distributor proteins. Various studies are available in the literature describing methods to test competitive binding of chemicals to TTR and/or TBG. Lans et al. (1993) tested environmental contaminants by using an in vitro TTR competitive binding assay (Table 1). Human TTR and ^125^I-labelled T4 were added to experimental solutions with various concentrations of test items. The protein-bound fraction and free ^125^I-T4 were separated by size-exclusion chromatography and the radioactivity of both fractions were analysed to determine the competitive displacement of ^125^I-T4 from TTR. The same principle was used by Collet et al. (2020), who incubated TTR with unlabelled T4 and various concentrations of test items and separated free T4 from TTR-bound T4 by size-exclusion chromatography. The concentration of TTR-bound T4 was determined in the TRβ-CALUX reporter gene assay, based on the agonistic activity of T4 towards thyroid hormone receptor beta (TRβ). Although the assay does not require precautionary measures for working with radioactive material, the size-exclusion chromatography step and the consequent 24-h exposure period in the TRβ-CALUX reporter gene assay make the assay less suitable for high-throughput screening purposes (Collet et al. 2020). Alternatively, Marchesini et al. (2006) developed a surface plasmon resonance (SPR) biosensor based method that was used to determine relative binding potencies of chemicals compared to T4. The experiments were conducted by injecting the experimental solution over the sensor chip surface, which is not time-effective when testing a large panel of compounds. Montaño et al. (2012) and Ren and Guo (2012) introduced the use of fluorescence T4 probes (8-anilino-1-naphthalenesulfonic acid ammonium salt (ANSA) and fluorescein isothiocyanate (FITC)), respectively, instead of ^125^I-labelled T4 to the TTR/TBG-binding assay. The throughput of the FITC-T4 binding assay was further increased by Ouyang et al. (2017), who measured fluorescence intensity (FI) in 96-well plates rather than cuvettes, allowing more test items to be tested within a short time period. More recently, Hamers et al. (2020) tested contaminants found in dust, maternal serum and infant serum by using a TTR-binding assay with FITC-T4 using a lower incubation temperature (4 ºC) and a longer incubation time (2 h). A summary of the main characteristics of several developed TTR assays is given in Table 1.Three main experimental methods have been published on TBG binding, which were basically based on the reported TTR-binding assays mentioned above: (1) a radio-ligand TBG assay; (2) an SPR biosensor technique (Cheek et al. 1999; Marchesini et al. 2006); and (3) a fluorescence polarization (FP) method (Ren and Guo 2012).

**Table 1:** Experimental methods and corresponding experimental conditions of published TTR binding assays. (The determined Kd and Ki values represent the dissociation constants of ligand and inhibitors, respectively)

	Ligand and ligand concentrations	Protein and protein concentrations	Incubation temperature	Incubation time	Determined Kd values	Determined Ki values of T4	References
^125^I-labelled T4 TTR assay	^125^I labelled T4, 55 nM	Human TTR, 30 nM	4 °C	overnight	N.D	50 nM	Lans et al. (1993)
TTR-TRβ-CALUX	T4, 52 nM	Human TTR, 62 nM	Room temperature	1 h	N.D	N.D	Collet et al. (2020)
SPR biosensor based TTR assay	T4, 100 nM	Recombinant TTR, 18.2 nM	25 °C	10 min	9.2 ± 1.2 nM	13.7 ± 1.3 nM	Marchesini et al. (2006)
ANSA TTR binding assay	ANSA, 600 nM	Human TTR, 500 nM	4 °C	2 h	943 nM*	166 nM*	Montaño et al. (2012)
FITC-T4 TTR binding assay in cuvettes	FITC-T4, 100 nM	Human TTR, 200 nM	Room temperature	5 min	92 ± 4 nM	239 ± 12 nM	Ren and Guo (2012)
FITC-T4 TTR binding assay in 96 well plates	FITC-T4, 141 nM	Human TTR, 281 nM	Room temperature	5 min	261 nM	N.D	Ouyang et al. (2017)
Updated FITC-T4 TTR binding assay in 96 well plates	FITC-T4, 110 nM	Human TTR, 30 nM	4 °C	2 h	140 nM	32 nM	Hamers et al. (2020)

*N.D.* not determined

*Ka of ANSA-TTR and T4-TTR was reported: 1.06 × 10^6^ and 6 × 10^6^ L/mol. respectively; Kd/Ki were calculated from Ka by definition: Ki = 1/Ka

**Fig. 1:**
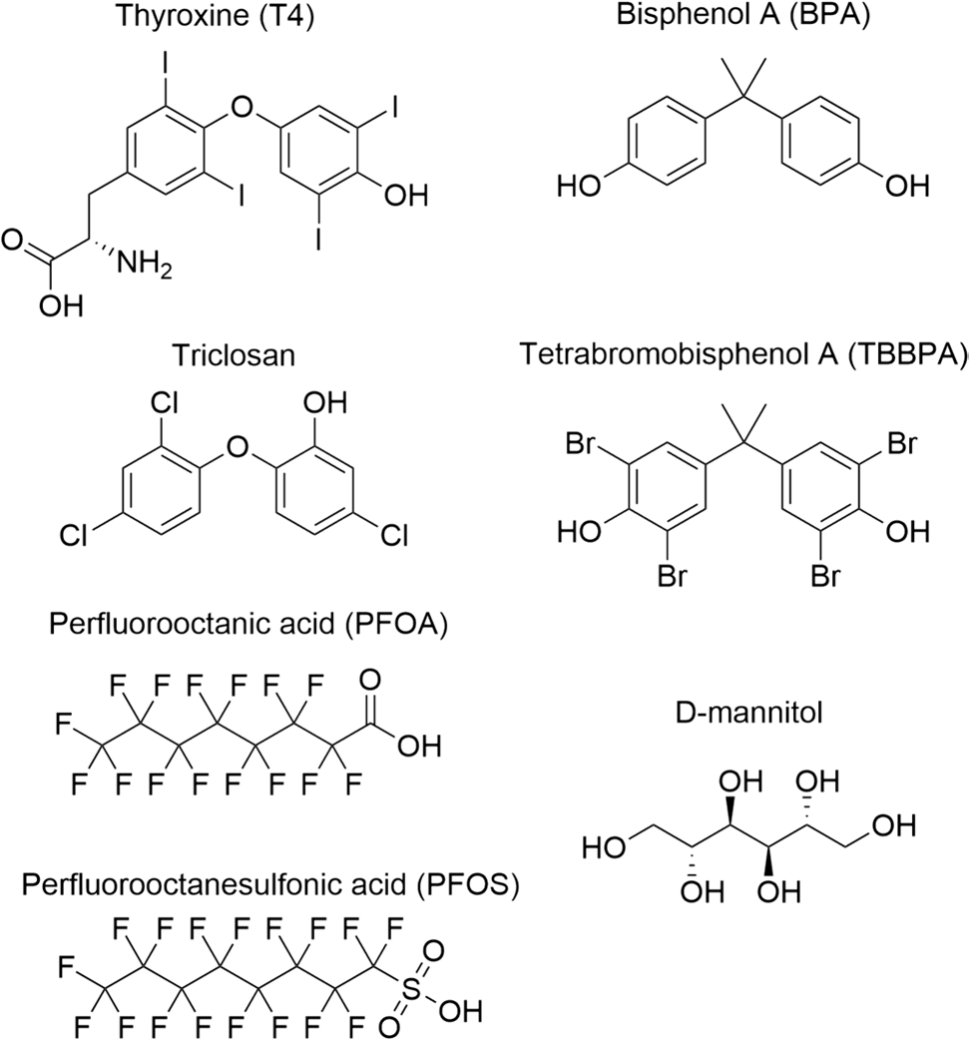
Reference compounds used for the TTR-binding assay and TBG-binding assay. D-mannitol served as a negative control

The goal of the present study was to develop optimized binding assays that are easy to perform, quick in use, and allow for a fast screening of chemicals for their capacity to compete with FITC-T4 for TTR- and TBG-binding. The TTR-binding assay was further optimized and pre-validated as part of an EURL ECVAM (European Union Reference Laboratory for alternatives to animal testing) study on the validation of 18 mechanistic non-animal methods that can detect THSD chemicals (Bernasconi et al. 2023). In addition, a new TBG-binding assay based on FP was developed. Regarding the TTR assay, temperature, incubation time and TTR and FITC-T4 concentrations were optimized and the optimized protocol was pre-validated with reference compound (T4) and six model compounds (Fig. 1). This optimized TTR protocol was also used as a base for the optimization of the FP based TBG-binding assay. First, Kd values were determined based on saturation curves observed from the binding of FITC-T4 to both proteins. Subsequently, the seven model compounds, including D-mannitol as a negative control, were tested in both the optimized TTR- and newly developed TBG-binding assays.

## Materials and methods

### Materials and reagents

Human TTR (prealbumin, > 95%) was ordered from Sigma Aldrich (Zwijndrecht, The Netherlands) and TBG (> 98%) from Bio-rad Laboratories, Inc. (Veenendaal, The Netherlands). Pyridine (anhydrous) (99.8%), triethylamine (> 99%), fluorescein 5-isothiocyanate isomer I (FITC) (> 90%), lipophilic sephadex, ammonium acetate (> 98%), ammonium bicarbonate (> 99.5%), sodium bicarbonate, tris(hydroxymethyl)aminomethane, sodium chloride, ethylenediaminetetraacetic acid (EDTA) (> 99%), dimethyl sulfoxide (DMSO) (> 99.5%), acetic acid (≥ 99.7%), sodium hydroxide (≥ 98%) were ordered from Sigma Aldrich. Hydrochloric acid (37%) was ordered from Actu-All Chemicals B.V. (Randmeer, The Netherlands). Thyroxine (3,3′,5,5′-tetraiodothyronine or T4) (≥ 98%), bisphenol-A (BPA) (≥ 99%), 2,2′,4,4′-tetrabromobisphenol-A (TBBPA) (97%), triclosan (certified reference material, TraceCERT^®^), perfluorooctanoic acid (PFOA) (98%) and D-mannitol (≥ 98%) were also ordered from Sigma Aldrich. Perfluorooctanesulfonic acid (PFOS) (95%) was ordered from SynQuest Laboratories, NC (Alachua, USA). The 96-well black chimney polystyrene non-binding plates were ordered from Greiner Bio-one B.V. (Alphen aan den Rijn, The Netherlands).

### Synthesis of the fluorescent probe

The procedure for preparation and characterization of the FITC-T4 conjugate is described in the Supplementary Information file (Section 3).

### Optimization and pre-validation of TTR-binding assay

The optimization of the TTR-binding assay was based on the assay first described by Ren and Guo (2012), downscaled by Ouyang et al. (2017) and further optimized by Hamers et al. (2020). To increase the convenience and efficiency of the assay, the incubation time and temperature, along with the concentration of the FITC-T4 concentration used by Hamers et al. (2020) were challenged. The improved experimental setup of the assay was subsequently pre-validated as part of the EURL ECVAM study (Bernasconi et al. 2023), where Wageningen Food Safety Research (WFSR) served as the validating laboratory being part of the European Union Network of Laboratories for the Validation of Alternative Methods (EU-NETVAL).

All TTR-binding experiments were performed in black 96-well polystyrene nonbinding plates. Experimental wells and background wells were prepared in triplicate per plate. Background wells contained different concentrations of FITC-T4 in the saturation experiments, or a combination of a fixed concentration of FITC-T4 with different concentrations of the compound of interest in the competitive binding experiments. So in both types of experiments, background wells did not contain TTR. Fluorescence intensity (FI) was measured at λ_ex_ = 485 ± 20 nm and λ_em_ = 528 ± 20 nm (CLARIOstar Plus microplate reader, BMG LABTECH).

To test the effect of the change in temperature and incubation time, a saturation experiment (*N* = 3, *n* = 3) was performed in Tris–HCl buffer (0.1 M Tris (Thermo Scientific, Rockford, USA), 0.1 M NaCl (Merck KGaA, Darmstadt, Germany), 1 mM EDTA (VWR International, B.V. Amsterdam, The Netherlands); pH 8.0). The concentration of TTR was fixed at 30 nM and the concentration of FITC-T4 was varied ranging from 0 to 1000 nM. Two 96-well plates were prepared at the same time. After shaking for 5 min on a plate shaker, one prepared plate was incubated on ice (at 4 ºC) and another plate was incubated at room temperature (22 ºC). For both plates the fluorescence was measured at different time points: 5 min, 15 min, 60 min and 120 min. Also, competition experiments with T4, PFOS and PFOA were performed at different temperatures and incubation times to study the effect of temperature and incubation time on the competitive binding ability (*N* = 3, *n* = 3).

The reference compound (T4) and six model compounds (BPA, TBBPA, triclosan, PFOS, PFOA, and D-mannitol, Fig. 1) were tested (*N* = 3, *n* = 3) with optimal experimental conditions. Dilution series of all compounds were prepared in DMSO, expected to have a concentration–response going from 0 to 100% relative fluorescence intensity, except for the expected negative compound D-mannitol. First, 48 µL Tris–HCl buffer (pH 8.0) was pipetted to the wells, followed by 2 µL of the compound (in DMSO). To the experimental wells 50 µL of 120 nM TTR (prepared in Tris–HCl buffer) was added (50 µL Tris-buffer to the wells for background measurement). Subsequently, 100 µL 220 nM FITC-T4 (prepared in Tris–HCl buffer) was added to all wells, resulting in a final volume of 200 µL in triplicate. Final solvent (DMSO) concentrations in all conditions amounted to 1%. All plates were shaken for 5 min on a plate shaker, followed by an incubation for 15 min at room temperature. The standard operation procedure (SOP) is provided in Supplementary Information file (Section 3).

### TBG-binding assay

Based on the optimized TTR-binding assay, FITC-T4 was adopted as fluorescent ligand for the development of a competitive TBG-binding assay. Rossi and Taylor (2011) published a protocol regarding the application of fluorescence polarization (FP) in measuring interactions between a fluorescent ligand and a protein. In the present study, FP was used as a readout in both saturation and competitive binding assays to determine the binding affinities of FITC-T4 with TBG and the inhibition levels of unlabelled chemicals because of the limited dynamic range obtained with an FI readout (“Development of a TBG-binding assay”). In contrast to saturation experiments using an FI readout, a fixed concentration of FITC-T4 is required for the determination of a saturation curve using an FP readout. The concentration of FITC-T4 was fixed at 5 nM and the concentration of TBG was varied ranging from 0 to 300 nM. The saturation experimental solution in each well was prepared with 25 µL Tris–HCl buffer, 25 µL 20 nM FITC-T4 solution in Tris–HCl buffer and 50 µL varying concentrations of TBG in Tris–HCl buffer, resulting in a final volume of 100 µL per well in duplicate. Prepared plates were first shaken for 5 min and then incubated for 15 min at room temperature before being analysed by the microplate reader at FP mode (CLARIOstar Plus, BMG LABTECH).

The same model compounds as used for the EURL ECVAM pre-validation of the TTR-binding assay were tested in the TBG-binding assay (T4: *N* = 5,* n* = 2; model compounds: *N* = 3,* n* = 3). Accordingly, dilution series of all compounds were prepared in DMSO. First, 24 µL Tris–HCl buffer was pipetted to the wells, followed by 1 µL of the compound stock solution. Then, 25 µL 20 nM FITC-T4 (in Tris–HCl buffer) and 50 µL 20 nM of TBG (in Tris–HCl buffer) were added to the wells, resulting in a total volume of 100 µL. Solvent (DMSO) concentrations in all conditions were 1%. The plates were shaken for 5 min on a plate shaker, followed by an incubation for 15 min at room temperature.

Fluorescence polarization was measured at λ_ex_ = 482 ± 16 nm and λ_em_ = 530 ± 40 nm (CLARIOstar Plus microplate reader, BMG LABTECH). The focus and gain for both channels were adjusted prior to measurement on wells containing 100 µL 5 nM FITC-T4. The adjusted polarization was set to a reference value of 35 mP. The adjustment gain values ranged from 1900 to 2200. The plate layout of TBG-binding assay is provided in Supplementary Information file (Section 4).

### Kd, IC_x_ and Ki calculations

Calculations of the IC_X_, Kd and Ki values of TTR-binding assays are described in the Supplementary Information file (Section 3). The Ki values reported in this study have been calculated based on IC_20_ results rather than IC_50_ values in case some compounds cannot reach IC_50_ effect level due to their lower potencies.

For the TBG-binding saturation experiment, a similar quadratic function was derived as for the TTR-binding saturation experiment (Supplementary Information, Section 3), but then adjusted for fluorescence anisotropy:

1 $$r= {r}_{L}+({r}_{C}{-r}_{L})\frac{\left({K}_{d}+\left[{P}_{T}\right]+\left[{L}_{T}\right]\right) - \sqrt{{\left({K}_{d}+\left[{P}_{T}\right]+\left[{L}_{T}\right]\right)}^{2}-4\times \left[{P}_{T}\right]\times \left[{L}_{T}\right]}}{2\times \left[{L}_{T}\right]}$$

with r being the measured anisotropy values, r_L_ the estimated maximum anisotropy of the free FITC-T4, and r_C_ the estimated minimum anisotropy of completely bound FITC-T4. [*L*_*T*_] is the total ligand concentration (5 nM FITC-T4) and [*P*_*T*_] is the total protein concentration ranging from 0 to 300 nM TBG in the saturation experiment. According to Eq. 1, parameters r_L_, r_C_ and Kd were estimated by a nonlinear regression between the measured anisotropy r and P_T_ in Graphpad.

For the competitive TBG-binding experiments, concentration–response curves were fitted:

2 $$r={r}_{min}+ \frac{\left({r}_{max}-{r}_{min}\right)}{1+{\left(\frac{IC50}{X}\right)}^{HillSlope}}$$

with *r*_*min*_ and *r*_*max*_ being the minimum and maximum anisotropy values, respectively. X is the concentration of added competitor. Equations that were used for the calculation of the IC_X_ and Ki values in the TTR-binding assays (Supplementary Information, Section 3) were also used to determine the IC_X_ and Ki values in TBG-binding assays without any changes, respectively. The single Kd, IC_20_, IC_50_ and Ki were determined based on replicates (*n*) in each experiment, and then the average and confidence intervals were determined based on the Kd, IC_20_, IC_50_ and Ki values from sperate experiments (*N*).

Differences in IC_20_, IC_50_ and Kd values were deemed statistically significant when the 95% confidence intervals (CI) of the calculated values based on the curves in GraphPad Prism did not overlap.

Alternatively, another approach to determine IC_X_, Kd and Ki values based on a theoretical model of ligand–protein binding biochemistry was taken as described in detail in the Supplementary Information file (Section 5). For this approach, R was used to fit the model to the data. The results obtained from the two approaches were compared in Section 3.4.

## Results and discussion

### Optimization of TTR-binding assay

To achieve a fast, high-throughput and cost-effective TTR competitive binding assay, experimental conditions were optimized aiming for an ultimate balance between proper functioning of the assay, physiological relevance, and practical ease. Therefore, first the impact was tested of different incubation temperatures (on ice (4 ºC) and room temperature (22 ºC)) and incubation times (5, 15, 60, and 120 min (at room temperature)) on the obtained Kd value for FITC-T4 and the Ki values of the selected test items. In a next step, different FITC-T4 concentrations (changing the FITC-T4:TTR ratio) were assessed.

**Fig. 2:**
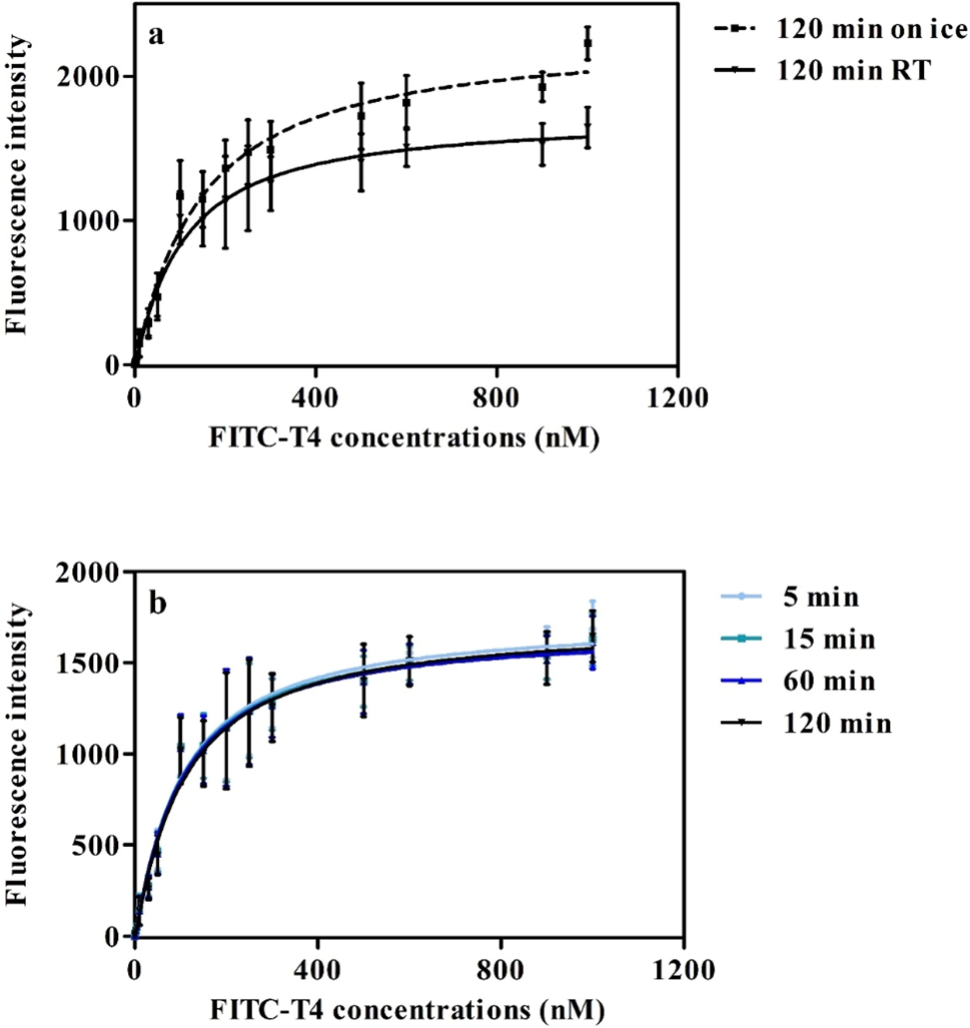
TTR saturation binding curves obtained with different experimental conditions. Assays were performed with 30 nM TTR at **a** different incubation temperatures (RT = room temperature) analysed after 120 min and **b** different time points at room temperature (*N* = 3, *n* = 3). Data are presented as the average (± SD) of separate experiments

The saturation curves of the FITC-T4-TTR complex at different incubation conditions and their corresponding Kd values are shown in Fig. 2 and Table 2, respectively. The Kd value of the FITC-T4-TTR complex at room temperature (143 nM, CI 127–159 nM) did not differ significantly from the Kd value on ice (157 nM, CI 124–190 nM), as the 95% confidence intervals overlap (Table 2). The Kd values of the FITC-T4-TTR complex also did not significantly change with different incubation times (Table 2). To demonstrate that differences in temperature and exposure time do not affect the outcomes for test items, competitive TTR-binding experiments were performed with T4, PFOS, and PFOA at different temperatures and incubation times (Fig. S1 and Table S1). For T4, PFOS, and PFOA, concentration response curves and corresponding Ki value estimations for competitive TTR-binding did not differ significantly between different incubation conditions.

**Table 2:** Kd values of the FITC-T4-TTR complex at RT and on ice after 120 min of incubation and at different time points at room temperature (RT). (CI = confidence interval, *N* = 3, *n* = 3)

Incubation temperature	Kd value (nM)	[95% CI of Kd]
On ice	157	[124–190]
RT	143	[127–159]
Incubation time at RT		
5 min	161	[130–191]
15 min	142	[124–161]
60 min	136	[121–150]

The single Kd was determined based on triplicates (*n* = 3) in each experiment, and then the average and confidence intervals were determined based on the three Kd values from sperate experiments (*N* = 3)

The results observed from both the saturation and the competitive binding experiments indicated that incubations on ice (at 4 ºC) can be replaced by incubations at room temperature, since no significant differences in the Kd and Ki values were obtained at two incubation temperatures. Kd and Ki values were not determined at 37 ºC, which may be considered more physiologically relevant. However, incubations at 37 ºC would make the protocol less suited for daily routine measurements as laboratories may lack plate readers with a heating element. Besides, testing at 37 ºC is not expected to provide more relevant data because it was reported that that FITC-T4 displacement from TTR by T4 at 37 ºC did not differ significantly from FITC-T4 displacement at 4 ºC or RT (Hamers et al. 2020). Additionally, the different incubation time points also did not significantly affect the Ki values or the binding potency of the test items. Although shorter incubation time (5 min) worked adequately, for practical reasons an incubation time of 15 min was chosen enabling easy screening of multiple plates by one technician (i.e., leaving sufficient time between different plates to perform the FI measurements).

In a next step, the impact was assessed of different FITC-T4 concentrations (11, 55 and 110 nM) in combination with 30 nM TTR (changing the TTR:FITC-T4 ratio) on competitive binding of T4 and PFOS (Fig. S2). The impact of the FITC-T4 concentration used is limited and using about 11 nM FITC-T4 still resulted in adequate curves. However, although the instrument used is sensitive enough to measure a low concentration of FITC-T4, instruments at other facilities might not be that sensitive. Therefore, in order to develop an assay that can be used at different labs, the concentrations of TTR and the FITC-T4 label were kept at 30 and 110 nM, respectively (i.e., the same as used before (Hamers et al. 2020)).

Eventually the assay turned out to work adequately at room temperature with an incubation time of 15 min and by using final concentrations of 30 nM TTR and 110 nM of the FITC-T4 label. Under these conditions, the Kd value of the FITC-T4-TTR complex is 142 nM (Table 2). The IC_50_ value of T4 with the FITC-T4-TTR complex is 125 (CI: 105–146) nM and the corresponding Ki value is 41 (CI 34–49) nM (Fig. 4, Table 3). These values are similar to IC_50_ = 100 nM and Ki = 32 nM, as previously reported (Hamers et al. 2020). These results demonstrate that the optimized protocol can be used for an easy, physiologically relevant, and fast in vitro testing of compounds for their capacity to disrupt T4 binding to TTR.

**Table 3:** IC_20_, IC_50_ and Ki values (nM) of the seven model compounds with TTR (*N* = 3, *n* = 3) and TBG (*N* = 5, *n* = 2 for T4 and *N* = 3, *n* = 3 for other compounds) from the present study and from the literature

Compound	TTR				TBG			
	IC_20_	IC_50_	Ki	Literature Ki*	IC_20_	IC_50_	Ki	Literature Ki*
T4	25 [22–29]	125 [105–146]	41 [34–49]	13.7 (Marchesini et al. 2006), 32 (Hamers et al. 2020), 50 (Lans et al. 1993), 166 (Montaño et al. 2012), 239 (Ren and Guo 2012)	9.2 [8.2–10.2]	14.9 [13.5–16.2]	2.5 [2–3]	2.2 (Qi et al. 2011), 7 (Ren et al. 2016), 17 (Ren and Guo 2012), 0.6 (Cao et al. 2010)
TBBPA	12 [11–13]	22 [20–25]	12 [10–14]	17 (Hamers et al. 2020)	7302 [5944–8661]	68,132 [62,074–74,190]	3762 [3062–4462]	N.A
Triclosan	436 [399–473]	1176 [1136–1217]	859 [796–921]	930 (Hamers et al. 2020)	284 [280–289]	1444 [1382–1506]	144 [142–147]	N.A
PFOS	118 [105–130]	271 [247–295]	243 [216–271]	160 (Hamers et al. 2020)	–	–	–	N.D. (Ren et al. 2016)
PFOA	267 [233–300]	763 [683–844]	568 [495–641]	1100 (Hamers et al. 2020), 60 (Ren et al. 2016)	–	–	–	N.D. (Ren et al. 2016)
BPA	14,716 [13,518–15,913]	63,060 [61,214–64,905]	32,137 [29,521–34,752]	3226 (Cao et al. 2011)	32,580 [30,290–34,871]	172,194 [150,126–194,262]	16,793 [15,612–17,974]	1695 (Cao et al. 2011)

Data are presented as the average with 95% confidence interval of data from separate experiments. The determination of IC_20_, IC_50_ in the TTR-binding assay was done according to the SOP in Supplementary Information Section 3.7.8. The IC_50_ of T4 in the TBG-binding assay was determined by a nonlinear concentration–response curve fitting according to Eq. 2. Ki values were derived from IC_20_ values similar as for TTR (Supplementary Information 3.8.1.11)

*N.D* means not detected, *N.A.* means not available

*Cao et al (2011) reported binding constants (3.1 × 10^5^ L/mol for BPA with TTR and 5.90 × 10^5^ L/mol for BPA with TBG), Ki calculated by Ki = 1/binding constant

### Development of a TBG-binding assay

Based on the optimized TTR-binding assay, a new TBG-binding assay was developed in the present study using FP as a readout. Initially, FI was used as a readout for the development of the TBG-binding assay to mimic the optimized TTR-binding assay as closely as possible. However, the saturation curve of the FITC-T4-TBG complex (30 nM TBG with different concentrations of FITC-T4) showed large variations for replicates within a single experiment (Fig. S3a). In addition, the signal to noise ratio (FI values of experimental wells divided by that of background wells) was very low and around 1.0 only (Fig. S3b). Upon binding to TBG, the increase in FITC-T4 was insufficient to distinguish TBG-bound from free FITC-T4, which may be explained by the different binding site of TTR and TBG for T4 (Refetoff 2023; Ren and Guo 2012). In order to increase the signal to noise ratio, a higher concentration of TBG (60 nM) was tested in the saturation experiment (Fig. S3b). With 60 nM TBG, the signal to noise ratio slightly increased compared to that with 30 nM TBG, but was considered not high enough to eventually obtain a robust TBG-binding assay using FI as readout. Therefore, FP was tested. It is considered as an alternative readout, as it has been demonstrated before to be a successful technique to study interactions between ligands and proteins (Rossi and Taylor 2011). In contrast to FI, where the emission intensity is measured, with FP the molecular Brownian rotation during the time between excitation and emission of the tracer is measured. FP is often expressed as anisotropy (*r*), which is defined as:

3 $$r=\frac{{I}_{\parallel }-{I}_{\perp }}{{I}_{\parallel }+{2I}_{\perp }},$$

with ${I}_{\parallel }$ the fluorescence intensity emission parallel and ${I}_{\perp }$ the fluorescence intensity emission perpendicular to the excitation direction. The basic principle of FP is that a small molecule, like unbound FITC-T4, rapidly rotates in solution resulting in a lower anisotropy, while a slower rotation is observed when FITC-T4 is bound to a larger molecule, like TBG in the present study, resulting in a higher anisotropy. When competitors are introduced to the FITC-T4-TBG complex, FITC-T4 is displaced, resulting in a decrease in anisotropy. Because the anisotropy signal is a weighted average of the bound and free FITC-T4 signals, saturation experiments with FP readout usually do not titrate increasing concentrations of ligand against a fixed concentration of protein, to avoid that the free ligand signal exceeds the signal of the bound ligand (Nosjean et al. 2006). Instead, saturation experiments are usually performed by titrating increasing concentrations of protein against a fixed concentration of ligand. Therefore, the saturation experiment for TBG had a different design than that for TTR: a fixed concentration of FITC-T4 and a range of concentrations of TBG were used.

**Fig. 3:**
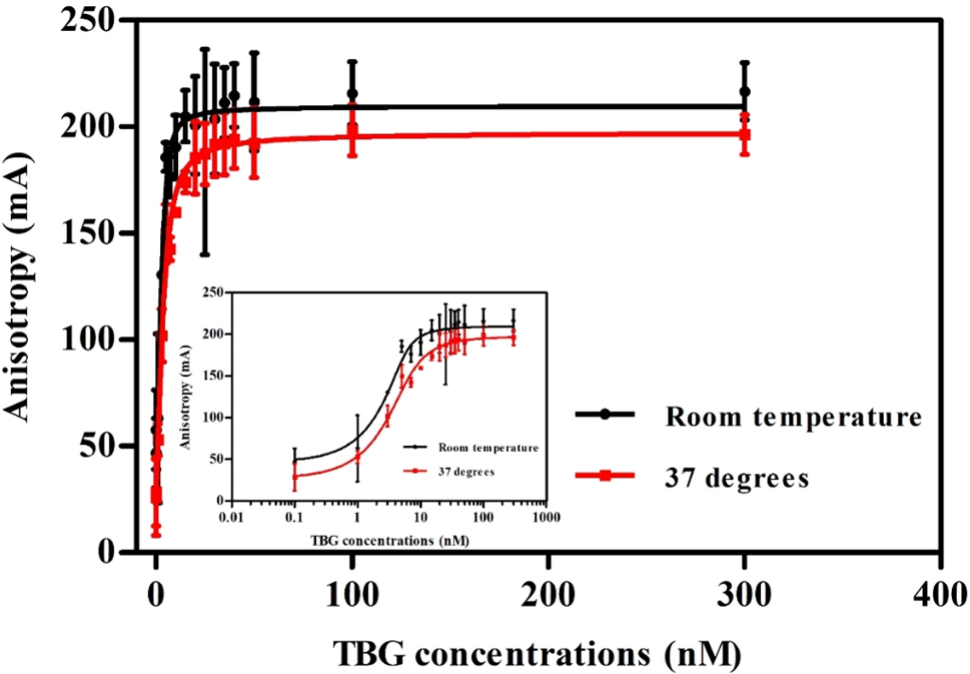
TBG saturation binding curves assays obtained with 5 nM FITC-T4 at two different temperatures (RT and 37 °C) (*N* = 2, *n* = 2, data are presented as the average (± SD) of separate experiments). The inserted graph shows the same data plotted with a logarithmic scale on the x-axis

Using 5 nM FITC-T4, the signal-to-noise ratio (FP values of wells to which 300 nM TBG was added divided by that of wells containing no TBG) of the saturation curve increased by as much as sixfold, indicating that for TBG the FP readouts have a wider dynamic range than FI readouts, allowing quantification of the binding capacity of potential competitors. In a preliminary TBG saturation experiment (Fig. 3) and competitive binding experiment (Fig. S4) two temperatures were tested: RT and 37 °C (to mimic the human in vivo situation) using an incubation time of 15 min. In this saturation experiment, the Kd value was determined to be 0.5 (CI 0.1–0.8) nM at RT and 1.3 (CI 0.4–2.2) nM at 37 °C, respectively (*N* = 2). Higher Kd values were observed with increasing temperature, which may be explained by the temperature-sensitive flexibility of the binding pocket (Qi et al. 2014, 2011). Concentration–response curves of test compounds T4 and triclosan did not significantly differ when tested at RT or 37 °C (Fig. S4, *N* = 2). Correspondingly, the Ki values at these two temperatures for T4 were estimated to be 2.9 nM (CI 0.2–5.6 nM) and 2.6 nM (CI 1.5–3.7 nM) respectively, and 192 nM (CI 178–206 nM) and 175 nM (CI 161–188 nM) for triclosan. Considering the small absolute difference in the Kd values and the similarity in concentration–response curves at the two different temperatures as well as practical concerns as described before for the TTR-binding assay, RT was also selected as the incubation temperature for the TBG-binding assay.

Saturation curves of the FITC-T4-TBG complex at RT were performed three times (Fig. S5) resulting in an average Kd value (CI) of the FITC-T4-TBG complex of 1.1 (CI 1.0–1.2) nM. In a next step, the concentration of TBG to be used in the competitive binding assay was selected. For competitive TBG binding studies, a concentration of TBG should be chosen that is high enough to bind a substantial amount of FITC-T4 before the addition of competitors (Huang 2003), but is low enough to avoid a large pool of free binding protein available for binding of competitors. Therefore, a concentration of 10 nM TBG was chosen.

**Fig. 4:**
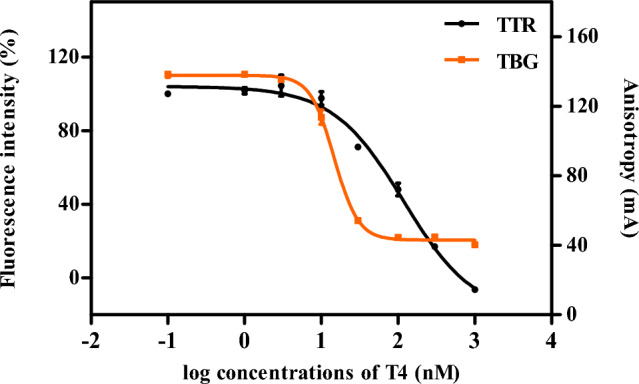
Concentration–response curves of T4 in the TTR-binding assay (left y axis, *N* = 3, *n* = 3) and in TBG-binding assay (right y axis, *N* = 5, *n* = 2). Data are presented as the average (± SD) of separate experiments

T4 had a higher potency to displace FITC-T4 from TBG than from TTR (Fig. 4). The IC_50_ value of T4 with TTR was 125 nM and that of T4 with TBG 14.9 nM (Fig. 4, Table 3). Correspondingly, the estimated Ki value of T4 with TTR (41 (CI 34–49) nM, *N* = 3, *n* = 3) was determined to be approximately one order of magnitude higher than that of T4 with TBG (2.5 (CI 2–3) nM, *N* = 5, *n* = 2). A higher binding affinity of T4 to TBG than to TTR was also reported and discussed by Ren and Guo (2012) who attributed this to structural differences and different properties of the ligand binding pockets of TTR and TBG.

### Seven model compounds tested in the optimized TTR-binding assay and in the newly developed TBG-binding assay

**Fig. 5:**
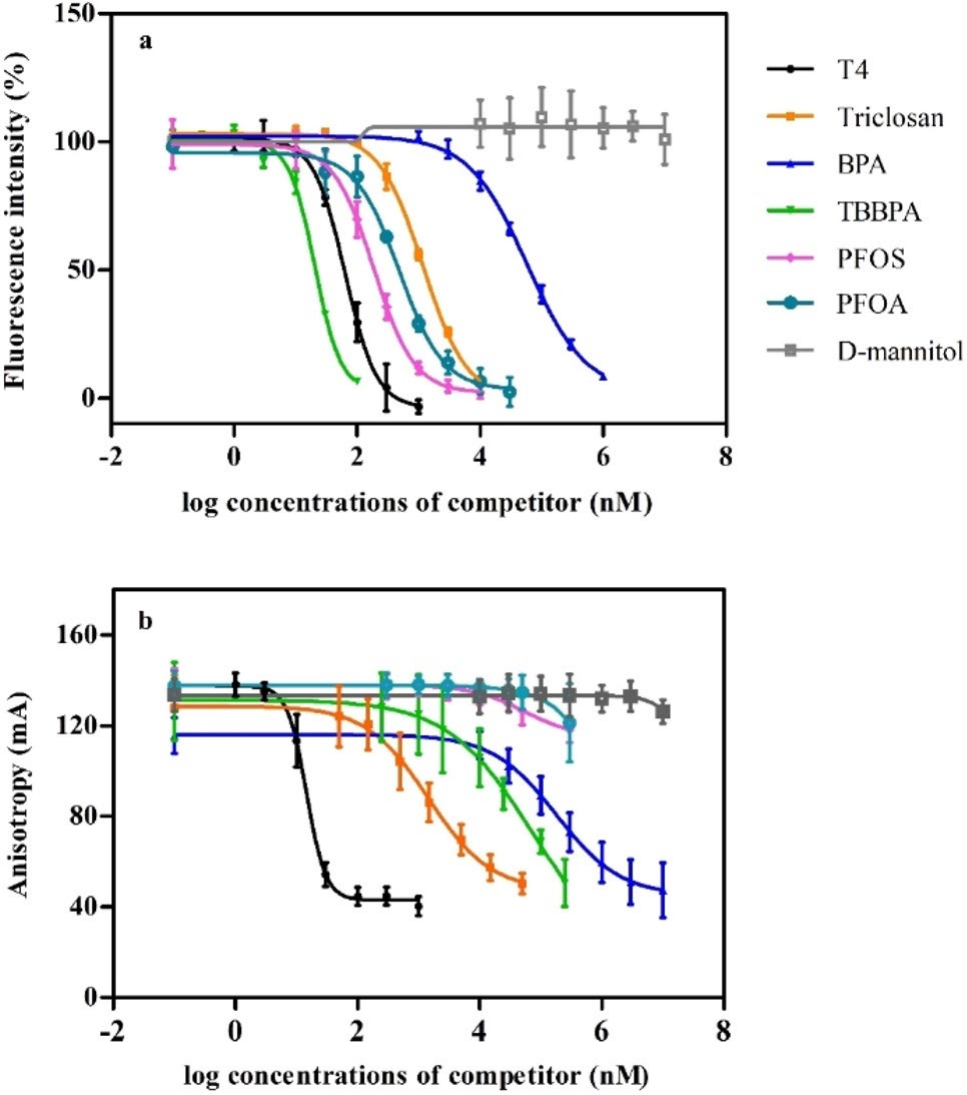
Concentration–response curves for seven model compounds **a** tested with 110 nM FITC-T4 and 30 nM TTR at RT (*N* = 3, *n* = 3); **b** tested with 5 nM FITC-T4 and 10 nM TBG at RT (*N* = 5, *n* = 2 for T4 and *N* = 3, *n* = 3 for other model compounds). Data are presented as the average (± SD) of data from separate experiments

To validate and compare the optimized TTR-binding assay and the newly developed TBG-binding assay methods, seven model compounds were selected and tested in both assays. Based on their previously reported competitive TTR binding the seven selected compounds were: T4 (as a positive reference chemical), D-mannitol (as a negative control), TBBPA, PFOS, PFOA, triclosan, and BPA (Collet et al. 2020; Weiss et al. 2015). The concentration–response curves of these compounds in the competitive TTR-binding assay are shown in Fig. 5a. Except for D-mannitol, all compounds displaced FITC-T4 from TTR with varying potencies (Table 3). TBBPA appeared to be the most potent compound (IC_50_ = 22 nM), and showed even a lower IC_50_ value than the endogenous thyroid hormone T4 (IC_50_ = 125 nM). The order of the competitive binding potency of the model compounds for TTR is: TBBPA > T4 > PFOS > PFOA > triclosan > BPA >  >  > D-mannitol. The corresponding IC_50_ and Ki values are shown in Table 3.

Figure 5b shows the concentration–response curves of the seven model compounds in the competitive TBG-binding assay. The negative control D-mannitol shows no inhibition of the FITC-T4 binding to TBG. No (or only limited) inhibition was observed for both PFOS and PFOA. Due to restricted solubility of both compounds in the assay medium, higher concentrations could not be tested. The model compounds showed displacement of FITC-T4 from TBG in the following order: T4 > triclosan > TBBPA > BPA >  >  > PFOA ≈ PFOS ≈ D-mannitol. The corresponding IC_50_ and Ki values are shown in Table 3.

### Evaluation of the assays and comparison of outcomes with the seven model compounds

The goal of the present study was to simplify and optimize the TTR-binding assay and develop a TBG-binding assay allowing easy, cheap and fast screening of chemicals for their binding capacity to TH distributor proteins. For both assays, similar Ki values of T4 were observed in the present and previous studies (Table 3). Results with seven model compounds showed that TBBPA, triclosan, PFOS, PFOA, and BPA compete with T4 for binding to TTR, which is in line with the results published by others (Cao et al. 2011; Cavanagh et al. 2018; Ren et al. 2016, 2020). In addition, the calculated Ki values of TBBPA, triclosan and PFOS are comparable to values published by others (Hamers et al. 2020), indicating that the updated experimental conditions (15 min, RT) do not affect the binding ability of the competitors to TTR to a great extent (Table 3).

According to different competitive binding experiments in the literature (Chi et al. 2020; Hamers et al. 2006) and this study, TBBPA binds more strongly to TTR than TTR’s natural ligand T4. While this is not the case for TBG, where T4 binds more strongly than TBBPA. Moreover, in the competitive TBG-binding assay, TBG demonstrated even a lower binding affinity (i.e., higher Ki) for TBBPA than TTR did (Table 3). Another FP-based competitive FITC-T4 binding assay with TTR and TBG showed binding of TBBPA to TTR, but not to TBG (Ren et al. 2020). In that study, the highest tested concentration was 100 µM TBBPA with 100 nM TBG and 50 nM FITC-T4 (Ren et al. 2020), whereas up to 250 µM TBBPA with 10 nM TBG and 5 nM FITC-T4 were used in the present study. These differences most probably result in a somewhat more sensitive screening, and therefore an effect of TBBPA in the TBG-binding assay in the present study.

BPA and its analogues have been reported to disturb the thyroid hormone system (Kim and Park 2019). BPA was the least potent chemical among the tested positive model compounds in both the TTR and the TBG assay (Fig. 5). Ki values of BPA for TTR- and TBG-binding derived from binding affinities reported by Cao et al. (2011) were one magnitude lower than those obtained in the present study (Table 3), indicating a weaker binding in the present study. In another study, however, Marchesini et al. (2006) observed no competitive binding to both TTR and TBG at the highest concentration of BPA tested (10 µM).

Triclosan was also reported to bind to TTR by using the ANSA probe and was also determined to be a more potent binder to TTR than BPA (Cavanagh et al. 2018). Few studies have investigated the binding capacity of triclosan with TBG. In a surface plasmon resonance biosensor assay with T4-coated chips and TBG, Marchesini et al. (2008) reported an IC_50_ value for triclosan of 1182 nM and for T4 of 17.2 nM. IC_50_ values from the present study were 1444 nM and 14.9 nM, respectively, being in line with the previous study. Direct comparison is difficult due to differences in test conditions and readout methods. Ki values for triclosan binding to TBG cannot be compared to previous studies, as to the best of our knowledge such Ki values have never been reported.

In agreement with previous findings by others, PFOS and PFOA bind to TTR but not to TBG (even when concentrations up to 1 mM were tested) (Ren et al. 2016). Most likely, PFOS and PFOA cannot adequately occupy the T4 binding pocket of TBG (Ren et al. 2016). Different binding affinities for TTR and TBG were not only reported for the compounds tested in the present study, but also for other compounds, including metabolites and derivatives of hydroxylated polychlorinated biphenyls (OH-PCBs) that bind stronger to TTR than to TBG (Cheek et al. 1999). These differences between TTR and TBG are most likely the result of differences in the binding pockets of TTR and TBG, i.e., hydrophobic channel T4 binding pockets in TTR and a surface binding pocket in TBG (Cao et al. 2011; Refetoff 2023). Hydroxylation, halogenation and presence of a hydroxylated phenyl ring has been reported to play crucial roles in the binding of chemicals to TH distributor proteins (especially the presence of an hydroxylated phenyl ring for binding to TBG) (Cao et al. 2010; Lans et al. 1994; Ren et al. 2016). This is confirmed by the data of the present study, as TBBPA, BPA, and triclosan all have these features (Fig. 1), while PFOS and PFOA miss a hydroxylated phenyl ring and do indeed not compete with T4 for binding to TBG. However, perfluorotridecanoic acid (PFTA) and perfluorotetradecanoic acid (PFTdA) that also lack a hydroxylated phenyl ring, were reported to bind to TBG with Ki values of 23 µM and 26.6 µM, respectively, which was explained with help of molecular docking that indicated that these longer fluorinated carbon chain acids fit the TBG binding pocket better than PFOA (Ren et al. 2016).

For the TTR-binding assay, the physiological relevance of the observed IC_20_ values for the model compounds was explored by a comparison with reported concentrations in human blood (Table S3). The TTR-binding capacity of a mixture consisting of the highest concentrations reported for the five TTR-binding model compounds in European biomonitoring studies was estimated to be equivalent to 19.1 nM of T4, according to the principle of concentration addition. The contribution of the different compounds to this T4-equivalent (T4EQ) concentration decreased in the order PFOS (50.9%) > triclosan (22.2%) ≈ TBBPA (19.5%) > PFOA (6.6%) > BPA (0.8%) (Table S3). Although the estimated 19.1 nM T4EQ only causes a 16% reduction of FITC-T4 binding to TTR in the bioassay, it is based on no more than concentrations of 5 xenobiotics in human blood. In a similar exercise with mixtures composed of maximum concentrations in human blood reported for 21 xenobiotics, the T4EQ concentration exceeded the IC_20_ in the assay by a factor of 3.5 (Hamers et al. 2020). In the same study, a mixture composed of median concentrations reported in human blood for the same 21 xenobiotics corresponded to 20% inhibition of FITC-T4 binding to TTR in the assay. Thus, the concentrations in human blood reported for individual xenobiotics may have a low TTR-binding capacity in the assay, but the combined mixture concentration of all TTR-binding xenobiotics in human blood gives a significant response. Similar estimation was also conducted based on the results of the TBG-binding assay (Table S4). The highest reported TBBPA, triclosan and BPA concentrations in blood in European countries were expressed in T4 equivalents, amounting to 2.2 nM of T4 equivalents, which is too low to displace T4 from TBG according to the concentration–response curve of T4. It must be noted, however, that one should be cautious when comparing nominal in vitro concentrations with total human blood concentrations, as there may be significant differences in concentration available for interaction with the targets (TTR and TBG) in vitro compared to in vivo. On the other hand, it should be realized that TTR is not only important as a distributor protein for TH in the blood, but also as a carrier protein for transporting TH across physiological barriers like the placenta or the blood-cerebrospinal-fluid barrier.

### Impact of concentration–response analysis on Kd, Ki and IC values

As mentioned in “TBG-binding assay”, different approaches were taken to estimate Kd and Ki values in TBG-binding assays. The first approach was a descriptive model. Curve fitting was done using Eq. 1 to estimate Kd and Eq. 2 to estimate a maximum and minimum anisotropy, the IC_50_, and the Hill slope in Graphpad. The second approach was based on a theoretical model that took into account the principles of fluorescence polarization and of biochemistry with respect to protein binding. Kd values were obtained by the same principle as the first approach. Ki values, however, were estimated by solving the model numerically in R, and were then converted into IC_X_ values. The idea behind taking two approaches was that the latter has a profound theoretical background, whereas the first is relatively easy to apply for users that are not experts in mathematical modelling, but rather prefer the use of vendor software like Graphpad. The equations used in theoretical model are described in the Supplementary Information file (Section 5) and the results obtained from the model are also shown in the Supplementary Information (Tables S2 and S5). The theoretically calculated Kd values were determined at 0.5 (CI 0.1–0.8) nM (22 °C) and 1.3 (CI 0.4–2.2) nM (37 °C) for temperature dependence experiments, and 1.1 (CI 1.0–1.2) nM at RT based on the repeated saturation experiments at RT (Table S2). As expected, these values are exactly the same as determined in Graphpad, because both methods use the same equation with least-squares fitting. Ki values estimated between both approaches were similar (Table S5).

## Conclusion

The experimental conditions for the TTR-binding assay were simplified and optimized for a fast and cost-effective chemical testing, and a new TBG-binding assay based on FP as a readout was developed. The comparison of dissociation constants between the previous TTR protocols and the newly optimized protocol confirmed that the updated protocol results in similar outcomes. Subsequently, seven model compounds were tested in both the updated TTR-binding assay and the newly developed TBG-binding assay to further assess both experimental methods. The tested compounds showed different binding characteristics for TTR and TBG, that were in line with previous observations by others. Both TTR and TBG are important TH distributor proteins in humans. The methods that were developed in the present study can be applied to screen xenobiotics for their binding affinity towards TTR and TBG, and outcomes can be used for prioritization for further (toxicity) testing (e.g., higher tier test systems) and/or for obtaining insight into the mode of action underlying TH disruption effects.

## Supplementary Information

Below is the link to the electronic supplementary material.

Supplementary file1 (DOCX 974 KB)

## Data Availability

Data will be made available on request.
