# Supplementary material for: Optimized methods for measuring competitive binding of chemical substances to thyroid hormone distributor proteins transthyretin and thyroxine binding globulin
Source: Arch Toxicol. 2024 Aug 21;98(11):3797–809. doi: 10.1007/s00204-024-03842-y (PMC11489250; doi:10.1007/s00204-024-03842-y)
Supplement: Supplementary file 1 — Supplementary file1 (DOCX 974 KB) [file 204_2024_3842_MOESM1_ESM.docx]

**Optimized methods for measuring competitive binding of chemical substances to thyroid hormone distributor proteins transthyretin and thyroxine binding globulin**

Yang Shen ^a^, Toine F.H. Bovee ^a*^, Douwe Molenaar ^b^, Yoran Weide ^a^, Antsje Nolles ^a^, Carmen Braucic Mitrovic ^b^, Stefan P.J. van Leeuwen ^a^, Jochem Louisse ^a, #^, Timo Hamers ^b^

^a^ *Wageningen Food Safety Research (WFSR), Wageningen University & Research, Akkermaalsbos 2, 6708 WB, Wageningen, The Netherlands*

^b^ *Amsterdam Institute for Life and Environment (A-LIFE), Vrije Universiteit Amsterdam, De Boelelaan 1085, 1081 HV, Amsterdam, the Netherlands*

^#^ *current affiliation: European Food Safety Authority (EFSA), Parma, Italy*

^*^Corresponding Author:

Toine F.H. Bovee

Wageningen Food Safety Research

Akkermaalsbos 2, 6708 WB Wageningen, The Netherlands

E-mail: toine.bovee@wur.nl

The supporting information file contains 5 sections:

| Section | Content | Page |
| --- | --- | --- |
| 1 | Supplemental figures | 3 |
| 2 | Supplemental tables | 8 |
| 3 | SOP TTR | 12 |
| 4 | Plate layout of TBG-binding assays | 34 |
| 5 | Theoretical model TBG-binding assay | 35 |

Section 1: Supplemental figures


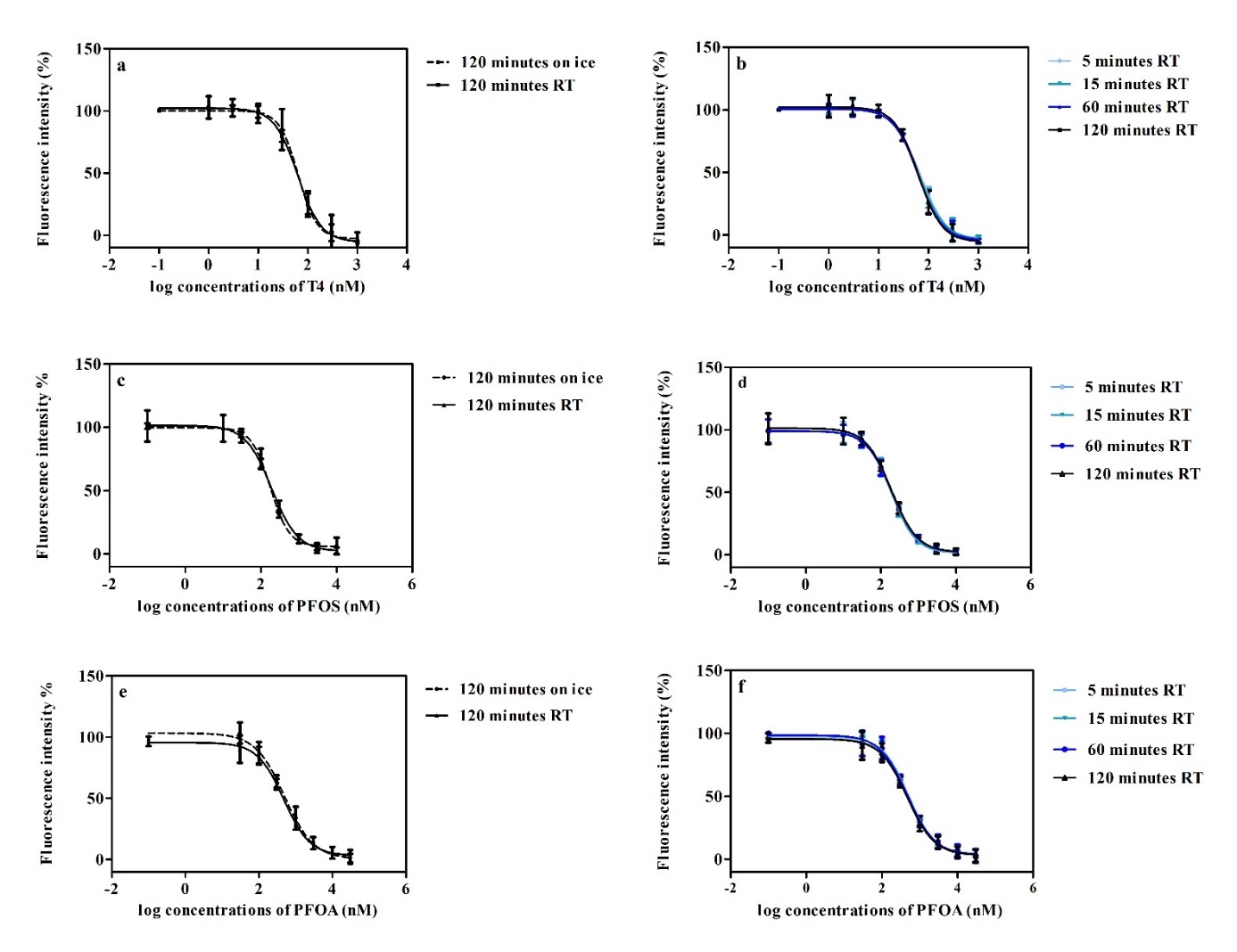
Figure S1. Concentration-response curves from competitive TTR-binding experiments (55 nM FITC-T4, 30 nM TTR) performed at room temperature (RT) and on ice for 120 minutes with (a) T4, (c) PFOS and (e) PFOA; and at RT for 5, 15, 60 and 120 minutes with (b) T4, (d) PFOS and (f) PFOA (*N* = 3, *n* = 3). Data are presented as the average (± SD) of triplicate experiments (*N* = 3).


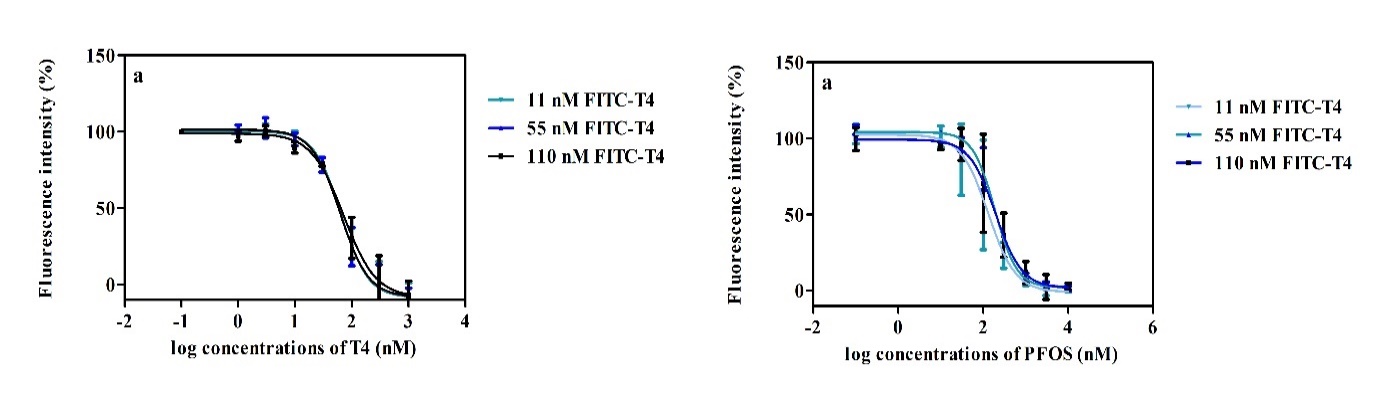


Figure S2. Concentration-response curves from competitive TTR binding experiments with (a) T4 and (b) PFOS were performed at varying concentrations of FITC-T4 for 5 minutes (*N* = 3, *n* = 3). Data are presented as the average (± SD) of triplicate experiments (*N* = 3).

Figure S3. (a) Saturation curves and (b) signal to noise ratios of 0-1000 nM titrated FITC-T4 into 30 nM or 60 nM TBG using FI as readout (*N* = 1, *n* = 3). Data are presented as the average (± SD) of triplicates (*n* = 3).

Figure S4. Concentration-response curves from TBG competitive binding assays with (a) T4 (*N* = 2, *n* = 2) and (b) triclosan (*N* = 2, *n* = 3) tested at two different temperatures. Data are presented as the average (± SD) of data from two individual experiments.

Figure S5. TBG saturation binding curve (5 nM FITC-T4) at room temperature. Data are presented as the average (± SD) of data from three individual experiments (*N* = 3).

Section 2: Supplemental Tables

Table S1. IC_50_ and Ki values (nM) of the FITC-T4-TTR (55 nM FITC-T4, 30 nM TTR) complex when incubated at RT (different time points) or on ice (120 min of incubation) (CI: confidence interval, *N* = 3, *n* = 3). The single IC_50_ and Ki were determined based on triplicates (*n* = 3) in each experiment, and then the average and confidence intervals were determined based on the three IC_50_ and Ki values from sperate experiments (*N* = 3).

| Incubation conditions | T4 | | PFOS | | PFOA | |
| --- | --- | --- | --- | --- | --- | --- |
|  | IC_50_ [95% CI] | Ki  [95% CI] | IC_50_  [95% CI] | Ki  [95% CI] | IC_50_  [95% CI] | Ki  [95% CI] |
| on ice  (120 min) | 66  [59-73] | 74  [47-101] | 179  [155-202] | 171  [142-201] | 461  [415-508] | 353  [280-426] |
| RT  (120 min) | 64  [59-69] | 53  [47-58] | 194  [172-216] | 133  [92-174] | 460  [432-487] | 262  [227-341] |
| RT  (60 min) | 63  [59-68] | 48  [45-51] | 196  [174-217] | 127  [104-150] | 459  [435-483] | 304  [267-341] |
| RT  (15 min) | 66  [62-69] | 50  [47-54] | 189  [165-212] | 133  [107-158] | 496  [472-520] | 280  [264-297] |
| RT  (5 min) | 72  [66-78] | 53  [49-57] | 191  [167-214] | 143  [113-173] | 500  [476-524] | 268  [221-315] |

Table S2. The estimated Kd values (in nM, 95% confidence intervals between brackets) of FITC-T4 to TBG using theoretical model. The single Kd was determined based on replicates (*n* = 2) in each experiment, and then the average and confidence intervals were determined based on the three Kd values from sperate experiments (*N*).

|  | RT (22 °C) | 37 °C |
| --- | --- | --- |
| Temperature optimization (*N* = 2, *n* = 2) | 0.5 [0.1-0.8] | 1.3 [0.4-2.2] |
| Repeated experiments (*N*= 3, *n* = 2) | 1.1 [1.0-1.2] |  |

Table S3: TTR-binding potency of highest concentrations reported for the model compounds in human blood.

| Model compound | TTR-binding assay response | |  | Concentration in human blood | | | | |
| --- | --- | --- | --- | --- | --- | --- | --- | --- |
|  | IC_20_ (nM) this study | Relative potency compared to T4 (T4-REP)^a^ |  | Highest concentration (nM) | Reference | T4EQ concentration^a^ (nM) | Contribution to T4EQ concentration (%) |  |
| TBBPA | 12 | 2.333 |  | 1.6 | Cariou et al. 2008^1^ | 3.73 | 19.5 |  |
| Triclosan | 436 | 0.064 |  | 66 | Allmyr et al. 2006^2^ | 4.24 | 22.2 |  |
| PFOS | 118 | 0.237 |  | 41 | Glynn et al. 2012^3^ | 9.73 | 50.9 |  |
| PFOA | 267 | 0.105 |  | 12 | Glynn et al. 2012^3^ | 1.26 | 6.6 |  |
| BPA | 14716 | 0.002 |  | 83 | Mielke et al. 2009^4^ | 0.16 | 0.8 |  |
| T4 | 25 | 1.00 |  |  |  |  |  |  |
| ΣT4EQ |  |  |  |  |  | 19.1^b^ |  |  |

^a^: For each test compound *i* holds T4-REP*_i_* = IC_20_(T4)/ IC_20_(*i*) and T4EQ*_i_* = (concentration)*_i_* x T4-REP*_i_*

^b^: The mixture has a TTR-binding potency equivalent to 19.1 nM T4, corresponding to a 16% inhibition in the binding assay.

Table S4: TBG-binding potency of highest concentrations reported for the model compounds in human blood.

| Model compound | TBG-binding assay response | |  | Concentration in human blood | | | | |
| --- | --- | --- | --- | --- | --- | --- | --- | --- |
|  | IC_20_ (nM) this study | Relative potency compared to T4 (T4-REP)^a^ |  | Highest concentration (nM) | Reference | T4EQ concentration^a^ (nM) | Contribution to T4EQ concentration (%) |  |
| TBBPA | 7302 | 0.001 |  | 1.6 | (Cariou et al. 2008) | 0.002 | 0.1 |  |
| Triclosan | 284 | 0.032 |  | 66 | (Allmyr et al. 2006) | 2.138 | 98.8 |  |
| BPA | 32580 | 0.0003 |  | 83 | (Mielke and Gundert-Remy 2009) | 0.023 | 1.1 |  |
| T4 | 9.2 | 1.00 |  |  |  |  |  |  |
| ΣT4EQ |  |  |  |  |  | 2.2^b^ |  |  |

^a^: For each test compound *i* holds T4-REP*_i_* = IC_20_(T4)/ IC_20_(*i*) and T4EQ*_i_* = (concentration)*_i_* x T4-REP*_i_*

^b^: The mixture has a TBG-binding potency equivalent to 2.2 nM T4.

Table S5. The estimated Ki values of four tested reference compounds in the TBG assay using a descriptive model in Graphpad and a theoretical model in R. (*N* = 5, *n* = 2 for T4 and *N* = 3, *n* = 3 for model compounds). The single IC_50_ and Ki were determined based on replicates (*n*) in each experiment, and then the average and confidence intervals were determined based on the three IC_50_ and Ki values from sperate experiments (*N*).

| Compounds | Ki values (nM) | | IC_20_ (nM) | |
| --- | --- | --- | --- | --- |
|  | Descriptive model | Theoretical model | Descriptive model | Theoretical model |
| T4 | 2.5  [2-3] | 0.8  [0.6-0.9] | 9.2  [8.2-10.2] | 5.9  [5.6-6.2] |
| TBBPA | 3762  [3062-4462] | 3308  [2613-4002] | 7302  [5944-8661] | 6421  [5083-7769] |
| Triclosan | 144  [142-147] | 149  [142-156] | 284  [280-289] | 293  [280-306] |
| BPA | 16793  [15612-17974] | 18424  [16808-20040] | 32580  [30290-34871] | 35744  [32609-38878] |

Section 3: SOP TTR

3.1 Objective and scope

This SOP describes the implementation of an in vitro TTR binding assay, in which items are tested for their potency to competitively inhibit the binding of fluorescently labelled thyroid hormone (FITC-T4) to its distributor protein TTR. In this SOP, test items are tested using as default solvent DMSO. Using other solvents to enhance solubility of the test item should only be considered when interferences of those solvents can be excluded as based on a solvent control check.

3.2 Definition

Thyroxine (T4) is a thyroid hormone, which is produced and released by the thyroid gland. Thyroxine-based hormones like T4 are primarily responsible for regulation of metabolism and play a major role in neurodevelopment. TTR is a distributor protein in serum that delivers T4 at its target (tissue). Thyroid hormone (TH) disrupting compounds are potentially important environmental contaminants due to their possible adverse neurological and developmental effects on both humans and wildlife. Affecting the binding of TH to TTR is regarded as one of the possible mechanisms of thyroid system disruption by chemicals.

3.3 Principle

Fluorescein isothiocyanate (FITC) is a fluorescent probe, which can be linked to T4. Upon binding of the FITC-T4 probe to human transthyretin (TTR) as a test system, an increase in fluorescence is observed most likely due to the elimination of intramolecular fluorescence quenching of the FITC group by the iodine groups of the bound T4. This increase in fluorescence, however, is abolished by adding competitors for TTR-binding like endogenous ligand T4 as a reference item or xenobiotic TTR-binding compounds as test items. When test items induce a concentration-dependent decrease in fluorescence in a FITC-T4/TTR competitive binding assay conform the results assessment, differences in potencies of these TH disrupting compounds can be determined by calculating the IC_50_ and/or Ki (inhibitory constant) values.

3.4 Test system, reference items, chemicals and reagents

3.4.1 Test system, reference items and label

1. Test system: Transthyretin (prealbumin, TTR) >95% from human plasma, CAS 87090-18-4, Sigma Aldrich (Zwijndrecht, The Netherlands)

2. Reference item: L-thyroxine (T4) >98%, CAS: 51-48-9, Sigma Aldrich (Zwijndrecht, The Netherlands)

3. Reference item: TBBPA 97%, powder, CAS 79-94-7, Sigma Aldrich (Zwijndrecht, The Netherlands)

4. Fluorescein 5-isothiocyanate isomer I (FITC) > 90%, CAS 3326-32-7, Sigma Aldrich (Zwijndrecht, The Netherlands)

3.4.2 Chemicals

1. Pyridine (anhydrous) 99.8%, CAS 110-86-1, Sigma Aldrich (Zwijndrecht, The Netherlands)

2. Triethylamine >99%, CAS 121-44-8, Sigma Aldrich (Zwijndrecht, The Netherlands)

3. Lipophilic Sephadex, CAS 9041-37-6, Sigma Aldrich (Zwijndrecht, The Netherlands)

4. Ammonium acetate > 98%, CAS 631-61-8, VWR International B.V. (Amsterdam, The Netherlands)

5. Ammonium bicarbonate >99.5%, CAS 1066-33-7, Sigma Aldrich (Zwijndrecht, The Netherlands)

6. Sodium bicarbonate, CAS 144-55-8, Fluka Analytical (Charlotte, USA)

7. Tris(hydroxymethyl)aminomethane, CAS 77-86-1, Thermo Scientific, Rockford, USA

8. Sodium chloride, CAS 7647-14-5, Merck KGaA (Darmstadt, Germany)

9. EDTA >99%, CAS 6381-92-6, VWR International, B.V. (Amsterdam, The Netherlands)

10. Ultrapure water

11. DMSO >99.5%, CAS 67-68-5, Sigma Aldrich (Zwijndrecht, The Netherlands)

12. Acetic acid ≥99.7%, CAS 64-19-7, Merck KGaA (Darmstadt, Germany)

13. Hydrochloric acid 37%, CAS 7647-01-0, Actu-All Chemicals (Randmeer, The Netherlands)

14. Sodium hydroxide ≥98%, CAS 1310-73-2, Sigma Aldrich (Zwijndrecht, The Netherlands)

3.4.3 Reagents preparation

3.4.3.1 Preparation of PWT (pyridine water triethylamine) mixture:

Mix 9 mL pyridine (3.4.2.1) with 1.5 mL ultrapure water (3.4.2.10) and 0.1 mL triethylamine (3.4.2.2), and mix well.

3.4.3.2 Preparation of NH4-acetate (0.2 M)

1. Weigh out 7.709 g ammonium acetate (3.4.2.4).

2. Dissolve in approximately 400 mL ultrapure water (3.4.2.10) in a 500 mL volumetric flask.

3. Adjust the pH to 4.0 with acetic acid (3.4.2.12).

4. Bring the volume to a total of 500 mL with ultrapure water.

3.4.3.3 Preparation of NH_4_HCO_3_ (0.05 M)

Dissolve 1.9765 g ammonium bicarbonate (3.4.2.5) in ultrapure water (3.4.2.10) in a 500 mL volumetric flask.

3.4.3.4 Preparation of NaHCO_3_ (0.05 M)

1. Dissolve 2.1005 g sodium bicarbonate (3.4.2.6) in approximately 400 mL ultrapure water (3.4.2.10) in a 500 mL volumetric flask (3.5.11).

2. Adjust the pH to 8.5 with 1 M NaOH (3.4.2.14).

3. Bring the volume to a total of 500 mL with ultrapure water.

3.4.3.5 Preparation of Tris-HCl buffer (0.1 M Tris, 0.1 M NaCl, 1 mM EDTA):

1. Weigh out 12.11 g Tris (3.4.2.7).

2. Weigh out 5.84 g NaCl (3.4.2.8).

3.Weigh out 0.372 g EDTA (3.4.2.9).

4. Dissolve the above substances in approximately 800 mL ultrapure water in a 1000 mL volumetric flask.

5. Adjust the pH to 8.0 with 1 M HCl (3.4.3.11).

6. Bring the volume to a total of 1000 mL with ultrapure water.

7. Storage life at room temperature: 2 months.

3.4.3.6 Preparation column packing Sephadex

1. Saturate Sephadex (3.4.2.3) overnight in ultrapure water (3.4.2.10) (1:10), i.e. 10 g Sephadex + 100 mL ultrapure water.

2. Store at 4°C (Storage life 3 years).

3.4.3.7 TTR stock solution (3.64 µM) in Tris-HCl buffer:

Careful: TTR is a tetramer protein and thereby a labile substance. Careful handling is required to prevent dimerization or monomerization of the protein. DO NOT VORTEX!

- Dissolve 1 mg TTR (3.4.1.1) in 5 mL cold (4°C) Tris-HCl buffer (3.4.3.5).

- Aliquot the stock solution in portions of 100 µL, store at -20°C. Storage life: 1 year.

3.4.3.8 TTR working solution (120 nM, single use):

- Add 90 µL TTR stock solution (3.4.3.7) to 2660 µL Tris-HCl buffer (3.4.3.5).

- Mix careful by homogenising the solution.

3.4.3.9 T4 stock solution (1000 µM) in DMSO:

- Dissolve 3.88 mg T4 (3.4.1.2) in 5 mL DMSO (3.4.2.11).

- Vortex briefly.

3.4.3.10 FITC-T4 working solution (220 nM):

- Pipette x volume of FITC-T4 produced in 3.7.1 and concentration determined in 3.7.1.3 into Tris-HCl buffer (3.4.3.5) to get 220 nM.

3.4.3.11 Hydrochloric acid (1M):

- Add 83 mL 37% hydrochloric acid (3.4.2.13) to 917 mL ultrapure water (3.4.2.10).

3.4.3.12 Sodium hydroxide (1M):

- Dissolve 40 g sodium hydroxide (3.4.2.14) in 1000 mL ultrapure water (3.4.2.10).

3.4.3.13 TBBPA stock solution (3000 µM) in DMSO:

- Dissolve 8.16 mg TBBPA (3.4.1.3) in 5 mL DMSO (3.4.2.11).

3.4.3.14 TBBPA working solution (300 µM):

- Add 300 µl of 3000µM TBBPA stock solution (3.4.3.13) to 2700 µL DMSO (3.4.2.11).

3.5 Equipment

3.5.1 Microplate reader (CLARIOstar Plus microplate reader, BMG LABTECH); with filter λ 485 ± 20 nm excitation and λ 528 ± 20 nm emission

3.5.2 UV-VIS cuvette-based spectrophotometer with shaker ɛ; 490 nm

3.5.3 96 Well black chimney polystyrene non-binding plates

3.5.4 Burette Column with Frit and Stopcock 300 mm x 10.5 mm ID x 13 mm OD

3.5.5 Plate shaker

3.5.6 Semi-micro cuvette (10 mm, suitable for and based on UV-VIS spectrophotometer)

3.5.7 Polypropylene tube, 50 mL

3.5.8 Centrifuge (for 50 mL polypropylene tube)

3.5.9 Polypropylene tube, 1.5 mL

3.5.10 Amber glass vials

3.5.11 Volumetric flask 500 mL

3.5.12 Volumetric flask 1000 mL

3.5.13 Clear glass vial

3.6 Safety precautions

Handling chemicals and test and reference items

Weighing and dissolving all chemicals and test and reference items should be done with care. Wear disposable gloves. Work in a safety cabinet or fume hood as much as possible. Materials that are contaminated with chemicals or test or reference items should be disposed in toxic waste. Store test and reference items in a fridge (-20°C and dark).

3.7 Procedure

General

Prior to testing test items for TTR binding potency the production and characterisation of FITC-T4 should be performed. Characterisation of the batch of the FITC-T4 is performed once after its production (7.1 and 7.2). After characterisation, the FITC-T4 should be aliquoted and stored, which then can be used for multiple separate runs (3.3.7.3 and 3.3.7.4).

3.7.1 Production and characterisation of a FITC-T4 batch for multiple separate runs

3.7.1.1 Preparing FITC-T4

1. Dissolve 10 mg FITC (3.4.2.3) in 0.5 mL PWT (3.4.3.1) in an amber glass vial (3.5.10): 51.4 mM FITC.

2. Dissolve 10 mg T4 (3.4.1.2) in 1 mL PWT (3.4.3.1) in an amber glass vial (3.5.10): 12.9 mM T4.

3. Mix both solutions (0.5 mL 51.4 mM FITC and 1 mL 12.9 mM T4) in a new amber glass vial (3.5.10) and incubate for 1 hour at 37°C.

4. Pipette the mixture (FITC + T4) into a new 50 mL polypropylene tube (3.5.7).


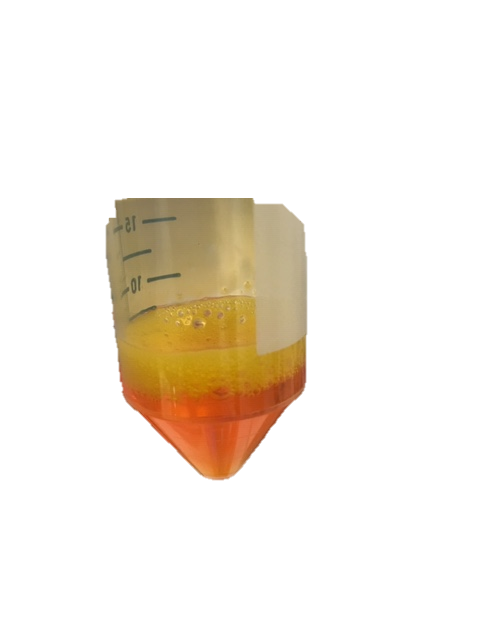
5. Precipitate the formed FITC-T4 by adding 20 mL of 0.2 M NH4-acetate (pH 4.0) (3.4.3.2).

6. Centrifuge for 10 min at 1000 g (3.5.8).

7. Discard supernatant.

8. Add 20 mL ultrapure water and mix vigorously.

9. Centrifuge for 10 min at 1000 g (3.5.8).

Figure S6-1: FITC-T4 before purification

10. Discard supernatant.

11. Dissolve the pellet in 5 mL of 0.05 M NH_4_HCO_3_ (3.4.3.3).

12. Mix thoroughly until FITC-T4 is dissolved (bright clear dark orange colour), see Figure S6-1 .

13. Optional: when mixture does not dissolve easily, incubate at 37°C for a few minutes.

3.7.1.2 FITC-T4 purification

Prepare a column of Sephadex in a burette:

1. Equilibrate the swollen Sephadex (3.4.3.6) to room temperature.

2. Resuspend/mix and then pour the Sephadex down with a glass rod into the burette (3.5.4).

3. Let the Sephadex settle and continue pouring until a 4.5 cm packing is achieved.

4. Equilibrate the Sephadex by passing 3 column volumes of 0.05 M NaHCO_3_ (3.4.3.4).

Purify FITC-T4 over the Sephadex-column:

5. Add 0.5 mL of the FITC-T4 (3.7.1.1) to the Sephadex-column.

6. Rinse with 10 column volumes 0.05 M NaHCO_3_ (3.4.3.4) (± 4.5 mL per column volume), Figures S6-2 and S6-3.

7. Elute pellet of interest (Figure S6-4) with 10 mL ultrapure water and collect the eluate temporarily in a 50 mL polypropylene tube (3.5.9)


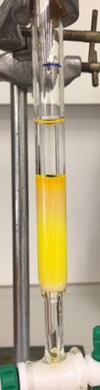

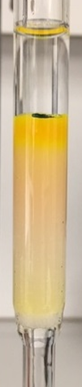

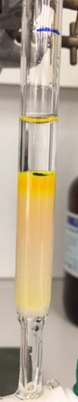


| Figure S6-2: After rinsing with 2 column volumes |  | Figure S6-3: After rinsing with 10 column volumes |  | Figure S6-4: Pellet of interest |
| --- | --- | --- | --- | --- |

3.7.1.3 Determination of the FITC-T4 concentration

When measuring the absorbance of FITC-T4 eluate, a small amount should be diluted by a factor of 10 with ultrapure-water.

1. Measure the absorbance of diluted eluate (FITC-T4) in a cuvette (3.5.6) with the UV-VIS cuvette-based spectrophotometer (3.5.2) and use ultrapure-water as a blank (the path length is 1 cm).

2. Calculate the concentration of FITC-T4:

$$Concentration FITC\text{-}T4 \left[ \frac{mol}{L} \right]=\frac{Ax-A0}{\varepsilon*l} *dilutionfactor$$

$$Ax=\text{absorbance of FITC-T4}$$

$$A0=\text{absorbance of blank (ultrapure water) }$$

$$l=\text{pathlength of cuvette in cm }$$

$$\varepsilon=molar extinction coefficient of FITC=7.8\cdot{10}^{4} M^{-1}{cm}^{-1}$$

3. Aliquot eluate (FITC-T4) preferably in volumes of 200 µL in a 1.5 mL polypropylene tube (3.5.9).

4. Store batch of FITC-T4 label at -80°C, concentration (3.7.1.3) and dissociation constant (3.7.1.4) should be retested after 5 years. Expiration time is not known.

3.7.1.4 Characterisation of the FITC-T4 label

A saturation curve should be performed to determine the dissociation constant (Kd) of the FITC-T4/TTR complex for characterisation of FITC-T4. This characterisation should be performed once after each newly produced batch of FITC-T4 label or retested after 5 years.

- Prepare a series of FITC-T4 working solutions based on the eluate from 3.7.1.2 and the calculated concentration in 3.7.1.3 with 15 different concentrations (1 to 15) and a blank (16) according to Table S4 by diluting it with Tris-HCl buffer (3.4.3.5).

|  | Concentration of FITC-T4 working solutions | Final concentration FITC-T4 in well |  | Concentration of FITC-T4 working solutions | Final concentration FITC-T4 in well |
| --- | --- | --- | --- | --- | --- |
| 1 | 2000 nM | 1000 nM | 9 | 200 nM | 100 nM |
| 2 | 1800 nM | 900 nM | 10 | 100 nM | 50 nM |
| 3 | 1200 nM | 600 nM | 11 | 60 nM | 30 nM |
| 4 | 1000 nM | 500 nM | 12 | 20 nM | 10 nM |
| 5 | 600 nM | 300 nM | 13 | 10 nM | 5 nM |
| 6 | 500 nM | 250 nM | 14 | 6 nM | 3 nM |
| e | 400 nM | 200 nM | 15 | 2 nM | 1 nM |
| 8 | 300 nM | 150 nM | 16 | blank (Tris-HCl buffer, 4.3.5) | 0 nM |

Table S4: Concentration series of FITC-T4 working solutions and a blank and their associated final concentrations per well in a 96 well plate (Figure S7).

- Pipette the following into a 96 well black chimney polystyrene non-binding plate (3.5.3) according to Figure S7 to get the final concentrations of FITC-T4 (3.7.1.2) as mentioned in Table S4:

With the addition of TTR per well (columns 1-3 and 7-9):

- 50 µL Tris-HCl buffer (3.4.3.5)
- 100 µL working solution x nM FITC-T4
- 50 µL 120 nM TTR working solution (3.4.3.8) (final concentration 30 nM)

Without addition of TTR per well (columns 4-6 and 10-12):

- 100 µL Tris-HCl buffer (3.4.3.5)
- 100 µL working solution x nM FITC-T4

Figure S7: Plate layout of the saturation experiment of FITC-T4/TTR-complex with final concentrations of FITC-T4 in nM.

After pipetting everything into the 96 well plate, subsequently:

1. Mix the plate by using the plate shaker (3.5.5) at 600 rpm for 5 minutes while keeping the plate in the dark.

2. Incubate for 15 minutes at room temperature while keeping the plate in the dark.

3. Measure the plate with the microplate reader (3.5.1), load the protocol for instrument setup (3.7.2) and make sure the light bulb is warmed-up. The measured fluorescence is given in relative fluorescence unit (RFU).

3.7.2 Instrument setup for fluorescent FITC-T4

1. Measure the filled 96 well plate (3.7.1.4) from the top.

2. Set the wavelengths at λ 485 ± 20 nm excitation and λ 528 ± 20 nm emission and use the appropriate filter(s) (cubes) or a linear variable filter monochromator for both excitation and emission.

3. The measured fluorescence range must fall within the dynamic range of the instrument (consult the instrument manual).

The gain setting of the instrument is only determined once per batch of FITC-T4 label during characterisation (3.7.1.4) and should remain the same for multiple runs of 3.7.4 and 3.7.5:

4. The gain setting should be fixed to a value that distinguishes the lowest concentration of FITC-T4 (1 nM) from the background (0 nM FITC-T4; blank; Tris-HCl buffer) with the following criteria (3.8.2.2):

RFU of 1 nM FITC-T4 subtracted by RFU of blank should be > +3SD of blank.

3.7.3 Process data to determine the dissociation constant (Kd)

For each FITC-T4 concentration, subtract the average fluorescence in the absence of TTR from the triplicate fluorescence values in the presence of TTR to obtain the fluorescence intensity (Y1):

$$Y1=\left[ RFU FITC\text{-}T4 with TTR \right]-\left[ mean RFU FITC\text{-}T4 without TTR \right]$$

- Make a two-column table with all tested ligand concentrations of FITC-T4 (L) and their corresponding fluorescence intensity values (Y1).

- Calculate the dissociation constant (Kd) by performing a non-linear regression on Y1 as a function of L according to the following quadratic equation (based on the assay conditions):

$$Y1 =constant*\frac{\left( K_{d}+30+L \right)-\sqrt{{(K_{d}+30+L)}^{2}-120*L}}{2}$$

used variables:

- 30 is the final concentration of protein (TTR) in nM (3.7.1.4)

- 120 is the concentration of protein (TTR) in nM multiplied by 4 (quadratic equation)

The required Kd value should be between 50 and 300 nM (3.8.2.1).

3.7.4 Preparation and pre-screening of reference and test items

After characterisation of FITC-T4 label multiple separate runs can be performed per batch.

All concentrations of reference and test items are prepared in DMSO (3.4.2.12).

3.7.4.1 Test Item stock solutions

1. Prepare a stock solution of test item by weighing a nominal amount (e.g. 25-50 mg/mL) and dissolving it in DMSO (3.4.2.12) into a clear glass vial (3.5.13) using a vortex. Note every procedural step for solving test items.

- N.B. using other solvents than DMSO to dissolve test items is not preferred, but if considered needed should be assessed for interference of this assay by adding a solvent control check (Figures S8 and S9).

- If not possible to solubilise, prepare a lower (e.g., 1:2) stock solution (or dilute existing stock) and check its solubility again.

2. Continue until the solution is soluble.

3. Record the concentration of the soluble test item stock solution.

3.7.4.2 Reference Item (T4) working solutions

1. Use the reference item (T4) stock solution (3.4.3.9) to prepare a calibration curve in the following work concentrations in amber glass vials (3.5.10) by diluting it in DMSO (3.4.2.11).

- 400 µM T4: pipette 480 µL of 1000 µM T4 (3.4.3.9) into 720 µL DMSO

- 100 µM T4: pipette 300 µL of 400 µM T4 into 900 µL DMSO

- 25 µM T4: pipette 300 µL of 100 µM T4 into 900 µL DMSO

- 6.25 µM T4: pipette 300 µL of 25 µM T4 into 900 µL DMSO

- 1.563 µM T4: pipette 300 µL of 6.25 µM T4 into 900 µL DMSO

- 390.6 nM T4: pipette 300 µL of 1.563 µM T4 into 900 µL DMSO

- 97.66 nM T4: pipette 300 µL of 390.6 nM T4 into 900 µL DMSO

- 0 µM T4: (blank; DMSO as solvent control (SC))

2. Prepare a plate according to 3.7.5 for each of the 8 concentrations of the reference item (3.7.4.2) using the plate layout as shown in Figure S8.

3.7.4.3 Range Finding (pre-screen) of test items

1. Prepare test item (pre-screening) serial dilutions from the test item stock solution (3.7.4.1) in the following work concentrations in amber glass vials (3.5.10) using DMSO (3.4.2.12) or other solvent.

- highest in DMSO soluble concentration of test item stock solution in molar or mg/ml when molecular weight is not known

- 10 x dilution test item: pipette 10 µL of test item stock into 90 µL DMSO, vortex briefly

- 10^2^ x dilution test item: pipette 10 µL of 10x dilution test item into 90 µL DMSO, vortex briefly

- 10^3^ x dilution test item: pipette 10 µL of 10^2^x dilution test item into 90 µL DMSO, vortex briefly

- 10^4^ x dilution test item: pipette 10 µL of 10^3^x dilution test item into 90 µL DMSO, vortex briefly

- 10^5^ x dilution test item: pipette 10 µL of 10^4^x dilution test item into 90 µL DMSO, vortex briefly

- 10^6^ x dilution test item: pipette 10 µL of 10^5^x dilution test item into 90 µL DMSO, vortex briefly

- 0 µM test item: (blank; DMSO as solvent control (SC), or other solvent)

2. Prepare 96 well plates according to 3.7.5, one 96 well plate with reference and a test item as displayed in Figure S8 and one or multiple plate(s) for test items as displayed in Figure S9. A plate with only test items like Figure S9 should include a plate control with a final concentration of 62.5 nM T4.

3. Visually check if test items are soluble in the Tris-HCl buffer (3.4.3.5) (microscopic observations may also be used for confirmation). Wells should be excluded in which insolubility is observed.

4. Perform the competitive TTR binding screening assay as described in 3.7.5 from point 5 through 3.7. Check if any binding competition is present and select a range of 7 possible concentrations that would provide a dose response (sigmoid) curve from 0 to 100% binding competition, which can be used for final testing (3.7.5). Do this by following the instructions in 3.7.8.1 and 3.7.8.2 and making a graph with the data from 3.7.8.2.

5. Repeat the previous 5 steps until a dose response (sigmoid) curve from 0 to 100% binding competition is found. Binding competition is present when the relative fluorescence intensity is equal to or lower than 100% at the lowest tested concentration and decreases with increasing concentrations of test item to a minimum of 0%.

3.7.5 The competitive TTR binding screening assay

After characterisation of FITC-T4 label multiple separate runs can be performed per batch.

1. Prepare serial dilutions of test items in DMSO, depending on potency and solubility as determined in 3.7.4. It should be pursued to have a dose response going from 0% to 100% relative fluorescence intensity (Y2; 3.8.1.8).

2. Pipette the following according to Figure S8 or Figure S9:

With addition of TTR (columns 1-3 and 7-9):

- 48 µL Tris-HCl buffer (3.4.3.5)
- 2 µL T4 reference item (according to 3.7.4.2 (Figure S8) or 6.25 µM (Figure S9)), 300 µM TBBPA (3.4.3.14) or test item [x] (take final concentrations into account)

Without addition of TTR (columns 4-6 and 10-12):

- 98 µl Tris-HCl buffer (3.4.3.5)
- 2 µL T4 reference item (according to 3.7.4.2 (Figure S8) or 6.25 µM (Figure S9)), 300 µM TBBPA (3.4.3.14) or test item [x] (take final concentrations into account)

3. Prepare a fresh 120 nM TTR working solution (3.4.3.8) (final concentration in the assay: 30 nM) and pipet directly to the plate according to Figure S8 or Figure S9:

- With addition of TTR (columns 1-3 and 7-9):
- 50 µl 120 nM of TTR working solution

4. Prepare a fresh 220 nM FITC-T4 working solution (30% extra volume) (3.4.3.10) (final concentration in the assay: 110 nM) and pipet directly to the plate according to Figure S8 or Figure S9:

- With and without addition of TTR (all wells):
  - 100 µl working solution 220 nM FITC-T4

FITC-T4 working solution should be prepared in excess with 30% extra volume that is minimally needed and should be added within 2 minutes after the preparation as “sinking” of FITC-T4 might appear:

Figure S8: Plate layout of the competitive binding experiment with T4 reference item, solvent control (SC), 3000 nM TBBPA as experiment control and test item, also referred to as reference plate. Note: concentrations are given in final concentrations per well.

Figure S9: Plate layout of the competitive binding experiment with 2 test items, solvent control (SC) and 62.5 nM T4 as plate controls. Note: concentrations are given in final concentrations per well.

5. After pipetting, shake the plate(s) on a plate shaker (3.5.5) at 600 rpm for 5 minutes on room temperature while keeping the plate(s) in the dark.

6. Incubate for 15 minutes on room temperature while keeping the plate(s) in the dark.

7. Measure the plate with the microplate reader (3.5.1), load the protocol for instrument setup (3.7.2) and make sure the light bulb is warmed-up. The measured fluorescence is given in relative fluorescence unit (RFU).

3.7.6 Check plate controls

3.7.6.1 Solvent control (IC_0_)

Determine the relative fluorescence intensity (RFI) of the solvent control (SC; IC_0_) on plate x (wells A1-A6 (Figure S9)) to the SC on the reference plate (wells A1-A6 (Figure S8)) as:

$$FI solvent control=\frac{\left[ mean RFU SC with TTR \right]plate x-\left[ mean RFU SC without TTR \right]plate x}{\left[ mean RFU SC with TTR \right]reference plate-\left[ mean RFU SC without TTR \right]reference plate}*100\%$$

This should be between 65 and 135%.

3.7.6.2 Positive experiment control (IC_100_)

Determine the relative fluorescence intensity (RFI) of the positive experiment control (3000 nM TBBPA (IC_100_), wells A7-A12 (Figure S8)) on the reference plate as:

$$RFI 3000 nM TBBPA= \frac{\left[ mean RFU TBBPA with TTR \right]reference plate-\left[ mean RFU TBBPA without TTR \right]reference plate}{\left[ mean RFU SC with TTR \right]reference plate-\left[ mean RFU SC without TTR \right]reference plate}*100\%$$

This should be between -10 and 10%.

3.7.6.3 Positive control (IC_50_)

Determine the relative fluorescence intensity (RFI) of the positive control 62.5 nM T4 (IC_50_) on plate x (wells A7-A12 (Figure S9)) to the reference plate (wells E1-E6 (Figure S8)) as:

$$RFI 62.5 nM T4=\frac{\left[ mean RFU T4 with TTR \right]plate x-\left[ mean RFU T4 without TTR \right]plate x}{\left[ mean RFU T4 with TTR \right]reference plate-\left[ mean RFU T4 without TTR \right]reference plate}*100\%$$

This should be between 65 and 135%.

If one of the control conditions is not met, the experiment should be repeated.

3.7.7 Check for autofluorescence or quenching by the test item

1. Determine the Pearson correlation coefficient (r) for the values of “RFU test item without TTR” and the corresponding ^10^log-transformed concentrations of the test item. Include the RFU values for the solvent control in this correlation. Do not use a test item concentration of 0 for the solvent control, because ^10^log(0) is undefined. Instead, fill out a concentration 1000x smaller than the lowest test concentration of the test item.

2. Test if r significantly deviates from zero by performing a Student’s t-test, with t=r/s_r_ and with $s_{r}=\sqrt{\frac{1-r^{2}}{n-2}}$, with n as the number of observations (n=24, according to Figure S8 and Figure S9). If |t|≥t_0.05(2),n-2_, the test item significantly interferes with the readout of the experiment, making the experiment potentially invalid. In case of n=24, the critical value of t_0.05(2),n-2_ is 2.0739. (3.8.2.7).

3. Determine the slope factor of “RFU test item without TTR” and the corresponding 10log-transformed concentrations of the test item via linear regression (slope of background fluorescence). If the slope is not between -61.7 and 39.0 the test item significantly interferes with the readout of the experiment, making the experiment invalid.

4. First, check the t-value, if it fits the requirement that |t| < 2.0739, then the test item does not affect the readout of the experiment, and IC_50_ and Ki values may be determined. Second, if the data does not fit the requirement for the t-value, then check if the slope of the background fluorescence is between -61.7 and 39.0. If this requirement fits, then the test item does not affect the readout of the experiment, and IC_50_ and Ki values may be determined.

3.7.8 Processing data to determine IC_50_ and calculate Ki value

1. Determine the relative fluorescence intensity (RFI; Y2) [%] as (3.8.1.8):

$$Y2=relative fluorescence intensity= \frac{\left[ RFU FITC-T4 with TTR \right]treatment-\left[ mean RFU FITC-T4 without TTR \right]treatment}{\left[ mean RFU FITC-T4 with TTR \right] solvent control-\left[ mean RFU FITC-T4 without TTR \right] solvent control}*100\%$$

2. Make a two-column table with final concentrations (A) of the test item and their corresponding relative fluorescence intensity values (Y2).

3. Calculate the median inhibition concentration (IC_50_) by performing a non-linear regression on *Y2* as a function of *A* according to Hill equation (3.8.1.9):

$$Y2=Y_{min}+ \frac{\left( Y_{max}-Y_{min} \right)}{1+\left( \frac{{IC}_{50}}{A} \right)^{HillSlope}}$$

4. Based on the values for IC_50_ and HillSlope, ICx values can be calculated for any percentage (x) of inhibited FITC-T4 binding, according to (3.8.1.10):

$$ICx={IC}_{50}*\left( \frac{100-x}{x} \right)^{\frac{1}{HillSlope}}$$

The IC_50_ value of the T4 calibration curve should be between 40 and 140 nM.

5. Based on the ICx value, the dissociation constant of the inhibitor-TTR complex (Ki) can be calculated according to (3.8.1.11):

$$Ki=\frac{Kd*PLx*ICx}{Lx*PIx}-Px$$

PLx is the concentration of the protein-ligand complex (i.e. FITC-T4 bound to TTR) at x% inhibition, which can be calculated under the bioassay conditions as:

$$PLx=\left( \frac{100-x}{100} \right)*\frac{\left( Kd+140 \right)-\sqrt{\left( Kd+140 \right)^{2}-13200}}{2}$$

used variables:

- 140 is the sum of the total concentrations in the test system of protein and ligand (TTR + FITC-T4) in nM

- 13200 is the product of the total concentrations in the test system of protein ligand (TTR x FITC-T4) in nM^2^ multiplied by 4 (quadratic equation)

Lx is the concentration of free ligand FITC-T4 at x% inhibition, which can be calculated under the bioassay conditions as:

$$Lx=110-PLx$$

used variables:

- 110 being the total concentration in the test system of the ligand (FITC-T4) in nM

PIx is the concentration of the protein-inhibitor complex (i.e. test item bound to TTR) at x% inhibition, which can be calculated under the bioassay conditions as:

$$PIx=30-PLx*\left( \frac{Kd}{Lx}+1 \right)$$

used variables:

- 30 is the total concentration in the test system of protein (TTR) in nM

Px is the concentration of free protein TTR at x% inhibition, which can be calculated under the bioassay conditions as:

$$Px=\frac{Kd*PLx}{Lx}$$

3.8 Results

3.8.1 Calculations

3.8.1.1 FITC-T4 concentration determination with cuvette based UV-VIS spectrophotometer (3.7.1.3):

$$Concentration FITC\text{-}T4 \left[ \frac{mol}{L} \right]=\frac{Ax-A0}{\varepsilon*l} *dilutionfactor$$

$$Ax=\text{absorbance of FITC-T4}$$

$$A0=\text{absorbance of blank (ultrapure water) }$$

$$l=\text{pathlength of cuvette in cm}$$

$$\varepsilon=molar extiction coefficient of FITC=7.8\cdot{10}^{4} M^{-1}{cm}^{-1}$$

3.8.1.2 Fluorescence intensity (Y1) (3.7.1.4):

$$Y1= fluorescence intensity=\left[ RFU FITC\text{-}T4 with TTR \right]-\left[ mean RFU FITC\text{-}T4 without TTR \right]$$

3.8.1.3 Calculation of dissociation constant (Kd) for FITC-T4-TTR complex (3.7.1.4):

$$Y1 =constant*\frac{\left( K_{d}+30+L \right)-\sqrt{{(K_{d}+30+L)}^{2}-120*L}}{2}$$

3.8.1.4 Plate to plate solvent control (SC; IC_0_) (3.7.6.1):

$$RFI solvent control=\frac{\left[ mean RFU SC with TTR \right]plate x-\left[ mean RFU SC without TTR \right]plate x}{\left[ mean RFU SC with TTR \right]reference plate-\left[ mean RFU SC without TTR \right]reference plate}*100\%$$

3.8.1.5 Positive experiment control of 3000 nM TBBPA (IC_100_) (3.7.6.2):

$$RFI 3000 nM TBBPA= \frac{\left[ mean RFU TBBPA with TTR \right]reference plate-\left[ mean RFU TBBPA without TTR \right]reference plate}{\left[ mean RFU SC with TTR \right]reference plate-\left[ mean RFU SC without TTR \right]reference plate}*100\%$$

3.8.1.6 Plate to plate positive control of 62.5 nM T4 (IC_50_) (3.7.6.3):

$$RFI 62.5 nM T4=\frac{\left[ mean RFU T4 with TTR \right]plate x-\left[ mean RFU T4 without TTR \right]plate x}{\left[ mean RFU T4 with TTR \right]reference plate-\left[ mean RFU T4 without TTR \right]reference plate}*100\%$$

3.8.1.7 Check for autofluorescence or quenching by test item with Student’s T-test (3.7.7.2):

$$s_{r}=\sqrt{\frac{1-r^{2}}{n-2}}$$

3.8.1.8 Relative fluorescence intensity (RFI; Y2) (%) (3.7.8.1):

$$Y2=relative fluorescence intensity= \frac{\left[ RFU FITC-T4 with TTR \right]treatment-\left[ mean RFU FITC-T4 without TTR \right]treatment}{\left[ mean RFU FITC-T4 with TTR \right]solvent control-\left[ mean RFU FITC-T4 without TTR \right]e}*100\%$$

3.8.1.9 Concentration-response curve fit with IC_50_ calculation (3.7.8.3):

$$Y2=Y_{min}+ \frac{\left( Y_{max}-Y_{min} \right)}{1+\left( \frac{{IC}_{50}}{A} \right)^{HillSlope}}$$

3.8.1.10 ICx calculation (3.7.8.4):

$$ICx={IC}_{50}*\left( \frac{100-x}{x} \right)^{\frac{1}{HillSlope}}$$

3.8.1.11 Calculation of dissociation constant (Ki) for inhibitor-TTR complex (3.7.8.5):

$$Ki=\frac{Kd*PLx*ICx}{Lx*PIx}-Px$$

3.8.2 Results assessment

3.8.2.1 Kd value (3.7.1.4)

The Kd value of FITC-T4 should be between: 50 and 300 nM.

3.8.2.2 Instrument setup (3.7.2)

RFU of 1 nM FITC-T4 subtracted by RFU of blank (Tris-HCl buffer) should be > +3SD of blank.

3.8.2.3 Negative plate control (IC_0_) (3.7.6.1)

Test plate (plate x) to reference plate (plate 1) solvent control should be between 65 and 135% relative fluorescence intensity.

3.8.2.4. Positive experiment control (IC_100_) (3.7.6.2)

Experiment control of 3000 nM TBBPA should be between -10 and 10% relative fluorescence intensity.

3.8.2.5 Positive plate control (IC_50_) (3.7.6.3)

Test plate (plate x) to reference plate (plate 1) control of 62.5 nM T4 should be between 65 and 135% relative fluorescence intensity.

3.8.2.6 Autofluorescence and quenching by test item (3.7.7)

The Pearson correlation coefficient should be determined with Student’s t-test and the t-value should be lower than 2.0739. The slope of the background fluorescence of the test item should be between -61.7 and 39.0. If both requirements are not met, the experiment is invalid.

3.8.2.7 IC_50_ value of T4 (3.7.8)

The IC_50_ of T4 should be between 40 and 140 nM.

Section 4: Plate layout of TBG-binding assays

The Tris-HCl buffer and FITC-T4 used in TBG-binding assays were prepared by following the procedure described in Section 3 (SOP TTR). The TBG-binding assay was newly developed and the full SOP is not available, since the TBG-binding assay is not part of the EURL ECVAM pre-validation study. The plate layout of saturation and competitive TBG-binding assays are shown below.

S4.1. The plate layout of saturation TBG-binding assays. The final TBG concentration ranged from 0-300 nM and the fixed concentration of FITC-T4 was added at 5 nM.

S4.2. The plate layout of competitive TBG-binding assays (PO = protein only (10 nM TBG): the background anisotropy values of the protein; PS = protein surplus (300 nM TBG and 5 nM FITC-T4): the highest anisotropy values; FO = FITC-T4 only (5 nM FITC-T4); SC = solvent control)

Section 5: Theoretical model to determine Kd, IC_X_ and Ki values in TBG-binding assays.

S5.1 Kd estimation

The equilibrium binding equation for binding of ligand molecules with free concentration, receptor $P$ with free concentration and receptor-ligand complex $C$ with concentration, and dissociation constant (Kd) is:

$CK_{d}=LP$ (S1)

If the total concentrations of ligand and receptor are $L_{T}$ and $P_{T}$then the following conservation relations hold:

$P_{T}=P+C$ (S2a)

$L_{T}=L+C$ (S2b)

In TBG-binding assays, FP was used a readout and anisotropy was used in calculations for mathematical convenience:

$r=\left( 1-f \right)r_{L}+ r_{C}= r_{L}+f(r_{C}-r_{L})$ (S3a)

$f= \frac{r-r_{L}}{r_{C}-r_{L}}$ (S3b)

with r being the measured values, $r_{L}$ is the anisotropy of the free FITC-T4, and $r_{C}$ is the anisotropy of the bound FITC-T4. $f$ was defined as the fraction of bound FITC-T4.

Substituting $f= \frac{C}{L_{T}}$ or $C= fL_{T}$ in equations S1 and S2, then:

$fL_{T}K_{d}=LP$ (S4)

$P= P_{T}- fL_{T}$ (S5a)

$L= L_{T}- fL_{T}$ (S5b)

Combine equation S5 to equation S4:

$fK_{d}-\left( 1-f \right)\left( P_{T}- fL_{T} \right)=0$ (S6a)

$f^{2}{L_{T}-f(P_{T}+L_{T}+K}_{d})+P_{T}=0$ (S6b)

This quadratic equation can be solved as

$f=\frac{\left( K_{d}+\left[ P_{T} \right]+\left[ L_{T} \right] \right) - \sqrt{\left( K_{d}+\left[ P_{T} \right]+\left[ L_{T} \right] \right)^{2}-4\times\left[ P_{T} \right]\times\left[ L_{T} \right]}}{2\times\left[ L_{T} \right]}$ (S7)

Substituting$f$ into equation S3a yields

$r= r_{L}+(r_{C}{-r}_{L})\frac{\left( K_{d}+\left[ P_{T} \right]+\left[ L_{T} \right] \right) - \sqrt{\left( K_{d}+\left[ P_{T} \right]+\left[ L_{T} \right] \right)^{2}-4\times\left[ P_{T} \right]\times\left[ L_{T} \right]}}{2\times\left[ L_{T} \right]}$, (S8)

S5.2 Ki and IC_50_ estimation

Models for two ligands, L and M are two ligands that compete for binding to P:

$CK_{d}=LP$ (S9a)

$C_{M}K_{i}=MP$ (S9b)

$P_{T}=P+C+ C_{M}$ (S10a)

$L_{T}=L+C$ (S10b)

$M_{T}=M+C_{M}$ (S10c)

Substituting equation S10c to S9b:

$C_{M}= \frac{M_{T}P}{K_{i}+P}$ (S11)

Substituting equation S11 to S10b:

$P_{T}=P+{fL}_{T}+ \frac{M_{T}P}{K_{i}+P}$ (S12)

Substituting $C={fL}_{T}$in equations S9a and S10b :

$P= \frac{f}{1-f} K_{d}$ (S13)

Combining equation S12 and S13:

$\left( 1-f \right)P_{T}-fK_{d}-f\left( 1-f \right)L_{T}- \frac{fM_{T}}{\frac{K_{i}}{K_{d}}+\frac{f}{1-f}}=0$ (S14)

To simplify the equation S14, $\alpha$ was defined as $\frac{K_{i}}{K_{d}}$ :

$\left( 1-f \right)P_{T}-fK_{d}-f\left( 1-f \right)L_{T}- \frac{fM_{T}}{\alpha+ \frac{f}{1-f}}=0$ (S15a)

$f^{2}{L_{T}-f(P_{T}+K}_{d}+L_{T}+\frac{M_{T}}{\alpha+ \frac{f}{1-f}} )+P_{T}=0$ (S15b)

Equations 15 have no closed-form solution for $f$, *i.e.* a solution expression $f$ as a function of the fixed parameters ($K_{d}$, $P_{T}$, $L_{T}$), varying inhibitor concentrations ($M_{T}$) and the parameter that needs to be fitted ($\alpha=\frac{K_{i}}{K_{d}}$). Therefore, we can also not express anisotropy $r$ as a function of these parameters and of the additional parameters that need to be fitted, $r_{L}$ and $r_{C}$ using equations 3. This means that we can not use a common (nonlinear) least squares approach because these need a closed form solution expressing $r$ as a function of all parameters. However, a function was constructed in R that yields $r$ as a numerical solution rather than an analytical solution using the uniroot.all function from the rootSolve R-package ((Soetaert 2009; Soetaert and Herman 2009)) when given values for all parameters and variables ($K_{d}$, $M_{T}$, $P_{T}$, $L_{T}$, $\alpha$, $r_{L}$ and $r_{C}$). The output of this function was then used as the input to a nonlinear least squares fitting function (gsl_nls from the R-package gslnls)(Chau 2023) to find the values for $\alpha$, $r_{L}$ and $r_{C}$ that yield the least sum of squared differences between observed anisotropies and anisotropies predicted by the model for varying inhibitor concentrations.

Equations S15 and S3 were solved by non-linear fitting based on the minimization of least squared differences between observed and predicted anisotropies.

To calculate ${IC}_{X}$, the fraction $\left( f_{0} \right)$ of bound ligand to the receptor in the absence of competitor was estimated by using equation S15a when $M_{T}$ = 0 and the only solution is:

$f_{0}= \frac{K_{d}+ P_{T}+ L_{T} - \sqrt{\left( K_{d}+ P_{T}+ L_{T} \right)^{2}-4P_{T}L_{T}}}{2}$ (S16)

At ${IC}_{X}$ we have, by definition $f= \frac{100-X}{100}$ $f_{0}$. Substituting $x=\frac{X}{100}$ and defining y= 1- $x$ = $1-\frac{X}{100}$, then $f= yf_{0}$. Substituting this in equation S14 and solving for ${IC}_{X}$:

${IC}_{X}= \frac{1}{yf_{0}} ( \alpha+ \frac{yf_{0}}{1-yf_{0}})(\left( 1- yf_{0} \right)P_{T}-yf_{0}K_{d}-yf_{0}\left( 1-yf_{0} \right)L_{T})$ (S17)

**References**

Allmyr M, Adolfsson-Erici M, McLachlan MS, Sandborgh-Englund G (2006) Triclosan in plasma and milk from Swedish nursing mothers and their exposure via personal care products. Sci Total Environ 372(1):87-93 https://doi.org/10.1016/j.scitotenv.2006.08.007

Cariou R, Antignac J-P, Zalko D, et al. (2008) Exposure assessment of French women and their newborns to tetrabromobisphenol-A: occurrence measurements in maternal adipose tissue, serum, breast milk and cord serum. Chemosphere 73(7):1036-1041 https://doi.org/10.1016/j.chemosphere.2008.07.084

Chau J (2023) gslnls: GSL Nonlinear Least-Squares Fitting. In. <https://github.com/JorisChau/gslnls>

Glynn A, Berger U, Bignert A, et al. (2012) Perfluorinated alkyl acids in blood serum from primiparous women in Sweden: serial sampling during pregnancy and nursing, and temporal trends 1996–2010. Environ Sci Tech 46(16):9071-9079 https://doi.org/10.1021/es301168c

Mielke H, Gundert-Remy U (2009) Bisphenol A levels in blood depend on age and exposure. Toxicol letters 190(1):32-40 https://doi.org/10.1016/j.toxlet.2009.06.861

Soetaert K (2009) Nonlinear Root Finding, Equilibrium and Steady-State Analysis of Ordinary Differential Equations. R package 1.6.,

Soetaert K, Herman PM (2009) A practical guide to ecological modelling: using R as a simulation platform, vol 7. Springer
